# Effect of early administration of tetracosactide on mortality and host response in critically ill patients requiring rescue surgery: a sensitivity analysis of the STOPSHOCK phase 3 randomized controlled trial

**DOI:** 10.1186/s40779-024-00555-2

**Published:** 2024-08-19

**Authors:** Giorgio Noera, Alfio Bertolini, Laura Calzà, Mercedes Gori, Annalisa Pitino, Graziella D’Arrigo, Colin Gerard Egan, Giovanni Tripepi

**Affiliations:** 1 Health Ricerca e Sviluppo, Global Contractor for STOPSHOCK National Plan of Military Research Ministry of Defence, Rome, 00187 Italy; 2 Department of Medicine and Division of Clinical Pharmacology, School of Medicine, UNIMORE, Policlinico, Modena, 41124 Italy; 3 IRET Foundation, Ozzano Dell’ Emilia, Bologna, 40064 Italy; 4 Institute of Clinical Physiology (IFC-CNR), Section of Rome, Rome, 00185 Italy; 5 National Research Council-Institute of Clinical Physiology, Reggio Calabria, 89124 Italy; 6 CE Medical Writing SRLS, Pisa, 56021 Italy

**Keywords:** Critical care, Melanocortin, Cytokine, Mortality, Survival, Bleeding, Transfusion

## Abstract

**Background:**

Undifferentiated shock is recognized as a criticality state that is transitional in immune-mediated topology for casual risk of lethal microcirculatory dysfunction. This was a sensitivity analysis of a drug (tetracosactide; TCS10) targeting melanocortin receptors (MCRs) in a phase 3 randomized controlled trial to improve cardiovascular surgical rescue outcome by reversing mortality and hemostatic disorders.

**Methods:**

Sensitivity analysis was based on a randomized, two-arm, multicenter, double-blind, controlled trial. The Naïve Bayes classifier was performed by density-based sensitivity index for principal strata as proportional hazard model of 30-day surgical risk mortality according to European System for Cardiac Operative Risk Evaluation inputs-outputs in 100 consecutive cases (from August to September 2013 from Emilia Romagna region, Italy). Patients included an agent-based TCS10 group (10 mg, single intravenous bolus before surgery; *n* = 56) and control group (*n* = 44) and the association with cytokines, lactate, and bleeding-blood transfusion episodes with the prior-risk log-odds for mortality rate in time-to-event was analyzed.

**Results:**

Thirty-day mortality was significantly improved in the TCS10 group vs. control group (0 vs. 8 deaths, *P* < 0.0001). Baseline levels of interleukin (IL)-6, IL-10, and lactate were associated with bleeding episodes, independent of TCS10 treatment [odds ratio (*OR*) = 1.90, 95% confidence interval (CI) 1.39–2.79; *OR* = 1.53, 95%CI 1.17–2.12; and *OR* = 2.92, 95%CI 1.40–6.66, respectively], while baseline level of Fms-like tyrosine kinase 3 ligand (Flt3L) was associated with lower bleeding rates in TCS10-treated patients (*OR* = 0.31, 95%CI 0.11–0.90,* P* = 0.03). For every 8 TCS10-treated patients, 1 bleeding case was avoided. Blood transfusion episodes were significantly reduced in the TCS10 group compared to the control group (*OR* = 0.32, 95%CI 0.14–0.73, *P* = 0.01). For every 4 TCS10-treated patients, 1 transfusion case was avoided.

**Conclusions:**

Sensitivity index underlines the quality target product profile of TCS10 in the runway of emergency casualty care. To introduce the technology readiness level in real-life critically ill patients, further large-scale studies are required.

**Trial registration:**

European Union Drug Regulating Authorities Clinical Trials Database (EudraCT Number: 2007-006445-41).

**Supplementary Information:**

The online version contains supplementary material available at 10.1186/s40779-024-00555-2.

## Background

Critical care is a complex field of medicine, particularly due to its diversity and unpredictability [1]. In patients who were admitted for emergency, mortality rates are usually high and often associated with the presence of comorbid diseases [2]. Consequently, research in this area is complex due to the patients’ overlapping conditions that impact upon explanatory variables in key critically ill subgroups [3]. Many clinical trials in this setting have therefore failed, and to date almost no drug has been developed. Only learning from previous experience, trials may be subsequently revised by mapping model inputs and outputs in the simultaneous determination of the global sensitivities of parameters and structure [4].

An extreme target population includes patients’ criticality in National Early Warning Score 2 (NEWS2) system (NEWS2 score ≥ 7) as an independent global prognostic factor, for who have received a color code for emergency-rescue in triage call [5]. The unified version of severity for mortality risk stratification is the homogeneous scoring classification for track and trigger system to assess illness severity, risk of deterioration that requires emergency intervention without delay [6]. In these patients, consumption coagulopathy of blood clotting disorders occurs in 25–35% of patients hospitalized; if present, the associated rescue surgery markedly increases the incidence of blood loss, transfusion, and multi-organ failure as a cause of in-hospital mortality [7].

Historically, the pooled patients 30-day mortality in advanced life support is 10.4% in out-hospital and 31.3% for in-hospital [8]. In contrast, survival at hospital discharge is 26% and only 16% of patients present an assisted cardiac arrest [9]. The demographic assessment by priority color code in every country region is a representative estimate of the life-threatening state in the worldwide population by phenotype-genotype, gender, race, and ethnic distribution within country different weight and annual rate by hospital area [10]. The benchmark and impact within the European population can be provided by the Vast Area of Romagna district in Italy, that covers a functional area of 1,527,551 inhabitants for the National Health System in Italy. The annual mortality rates, years of life lost, and disability-adjusted life-years for 100,000 inhabitants are about 319 years for emergency-rescue surgery, and are red color-coded (i.e., red-coded) [11]. The regionalization policy for cardiovascular surgical procedures for 1 million inhabitants is based on 1000 procedures, of which 15–18% must be performed for red-coded workups at one hub hospital [12].

The European System for Cardiac Operative Risk Evaluation (ES2) is the basis for hospital performance, which is recorded in medical records and administrative databases [13]. The tools for ES2 are based on prior-risk log-odds of conjugate light-tailed metanalysis of calibrated observed-to-expected (O/E) 30-day mortality rate in *U*-statistic for 18 Z-score strata by area under the receiver operating characteristic (ROC) curve [14]. The tracking of red-coded in ES2 (Z6, Z16) represents a default instant risk of > 14% at bedside with sensitivity of 70.73% and specificity of 81.39% in prediction accuracy of Z-area under ROC curve (Z-AUC) [15].

Pre-clinical studies over the past 3 decades have defined the potential of melanocortins [particularly adrenocorticotropic hormone (ACTH) 1–24, i.e., tetracosactide or TSC10 and α-melanocyte-stimulating hormone (α-MSH)] to regulate septic shock, extreme inflammatory activity and autoimmune disease [16–21].

Evidence from other preclinical as well as clinical studies highlights their effectiveness in restoring cardiovascular and respiratory function, and improving survival in conditions of systemic inflammatory response such as circulatory shock [22–25]. Given the chemotaxis of melanocortin as a strong anti-inflammatory anisotropy, the official classification and nomenclature is approved for human target drug in Class A by the Food and Drug Administration (FDA) and European Medicines Agency (EMA) for selective inhibition of nuclear factor kappa-B (NF-κB) and pro-inflammatory cytokines [26, 27].

This study aimed to evaluate the effect of the melanocortin TCS10 in critically ill patients in improving survival and coagulopathy control in surgical rescue. The association between changes in levels of pro-inflammatory cytokines and these outcome measures were assessed by Bayesian sensitivity approach.

## Methods

### Study design and participants

The Defense Industry Agency, Military Pharmaceutical Branch, NAGE AR054 and National Council of Research were responsible for establishing the quality target product profile as represented in the International Council for Harmonization (ICH) Q8/Q9/Q10 guidelines (EMA/CHMP/ICH/902964/2011). The study was compliant to National Bioethics Committee and Ethics Committee for informed consent due to the urgency of their situation, caused by sudden life-threatening or other sudden serious medical conditions approved under the accordance of Declaration of Helsinki, ICH for pharmaceuticals human drug use and good clinical practice and governmental quality audits (CEAV Prot 1841/2013 I.5/30).

This was a randomized, two-arm, multicenter, double-blind, controlled trial with the aim to evaluate the effect of TCS10 (the name of the investigational melanocortin product/agent) in improving survival and coagulopathy control in surgical rescue. The study was undertaken from April to August 2013, in the Emilia Romagna region, defined by the regional National Health System PSN 1998–2000 as a cluster of 4581 sequentially admitted patients to Hospital Hub code 080239, for cardiovascular event of which 459 underwent surgery. The target population [randomized controlled trial (RCT)’s subgroup of 100 consecutive patients; 56 were assigned to TCS10 group (standard surgical approach + intravenous bolus injection of 10 mg TCS10) and 44 were assigned to control group (standard surgical approach)] included critically ill victims for incoming hospital admission or in-hospital surgical rescue for acute cardiovascular events or who have received an emergency surgical red-coded triage call (without informed consent) because they are in extremis of a life-threatening condition (Additional file 1: Fig. S1).

The referred condition is critical and emergency, as categorized by the ES2 Z9–Z16 scoring system adopted in the regional record database for the assessment of cardiac surgical risk through guidelines and consensus statements strategy of the European Society of Intensive Care Medicine. The driving design time-to-event was the likelihood of exact inference of Z-AUC for risk of the exposure to surgery (T_0_) and its schedule, set at 30 days (T_30-day_).

The sample size of the study endpoint was cross validated for superiority sample size in time to event by Cox proportional hazards regression model on additive covariates on survival time by using Med Calc^®^ Statistical Software version 20.022 (MedCalc Software Ltd., Ostend, Belgium) for categorical predictive function normally distributed in a 1:1 randomization [28]. The power log-rank test and Cox proportional hazards one-sided superiority was performed using a proportional hazards model. The quantitative covariate in strata had a type I error α of 0.025, type II error β of 0.14 (86% power); survival data control 0.80; arm 0.999; number of cases required 56 for both groups and a total sample size 112. The Makuch & Simon’ formula was confirmed by Cox proportional hazards one-sided superiority [29].

Inclusion criteria were adult male or female patients admitted to hospital for emergency cardiac surgery with a NEWS2 score ≥ 7 and log (Z-score) probabilistic ES2 distribution mortality rate ≥ 14% in principal strata. Patients were not excluded due to the presence of comorbid diseases or previous or current treatment. Patients satisfying inclusion/exclusion criteria were randomly assigned (1:1) to receive the standard surgical approach to treatment and critical care (control group, i.e., volume restoration and inotropic drugs) or the same treatment plus an intravenous bolus injection of 10 mg TCS10 at the moment of arrival in the casualty ward (i.e., emergency room) (TCS10 group).

Arrows and connecting covariates to causality in finite direct acyclic graph depicted the participants in the primary analysis as cardinals in time space [30]. These subjects were considered in the time-to-event model of a multi-state model for the accelerated failure of life expectancy [31, 32]. This sensitivity analysis was conducted under EMA/CHMP/ICH/436221/2017 Committee for Medicinal Products for Human Use, ICH E9 (R1) addendum principle for estimands in clinical trials guideline on statistical principles [33]. This trial was registered on the European Clinical Trials Register (EudraCT Number: 2007-006445-41; https://www.clinicaltrialsregister.eu/ctr-search/trial/2007-006445-41/IT).

### Randomization and blinding

This study was designed as a randomized, two-arm comparative trial with 1:1 allocation, with a fixed sample size that was pre-determined based on covariate of ES2 random block sizes and statistical considerations to obtain a definitive assessment of the treatment effect via pre-defined hypothesis testing. The ES2 covariate was considered a unified family of covariate adaptive randomization procedure. The 1:1 allocation was based on a computer-generated randomization list using electronic case report forms (eCRFs) and medical records of web-based clinical research management using Stata 9.0 (StataCorp, College Station, TX, USA) by an independent doctor. Randomization was based on medical record code and central pharmacy online warehouse by date that was not linked to the turnover of medical staff in the operating room and intensive care unit (ICU) for the date of the emergency operation.

TCS10 was administered (or not) in a single dose during surgery. This implied that group assignment was hidden from the participant. The clinical trial followed standard operating procedure (SOP) of randomized administration ampoules including those with TCS10’s and was restricted to ID-barcode without any signaling of processing of treatment tracked to it. The unique ID-barcode entering the data was read and transferred directly to a web server on specific string of the eCRF and pharmacy warehouse address that was blinded to medical staff. The digital records for final cross-checking by outcome assessor were between eCRFs and medical records in an external blind web platform. In addition, blinding procedures were carried out for the collection, storage outcome assessor and transportation in external laboratories for blood sample analysis of cytokines. The SOP was based on the impossibility of reading the type of treatment and the input of the analysis related to it. Data input values in the eCFR2 were with restricted access and password that allowed to enter data in a blinded fashion for numeric strings. The access control server on the eCRF2 was off-line from the information technology team as an independent structure, also blind to it during the trial. Therefore, both the clinical protocol timeline and SOP significantly reduced the possibility that participants or experimenters could have influenced the results.

### Treatment

TCS10 is a drug targeting melanocortin receptors (MCRs) as heterometric family of rhodopsin family of 7-transmembrane G-protein coupled receptors ligand-gated type of both the central nervous system and peripheral tissues signaling pathways [34, 35]. Biological plausibility in evidence base data is by the full MCR (MCR1–5) action kinetics by synthetic peptide which is identical to the 24-amino acid segment to the N-terminal of similar ACTH in all species. The powder solution is for injection dosage strength of 10 mg as critical gradient for modulating energy cell line [cyclic adenosine monophosphate (cAMP), calcium mobilization, and phosphorylation of extracellular protein kinases] [27]. This dosage was administered as a single bolus injection (i.v.) in patients in a 5 ml volume of saline.

The elimination half-life following i.v. injection of TCS10 is about 7 min in the first hour, about 37 min in the next hour and about 3 h in the terminal phase [36]. The potency dose-strength was calculated on maximal tolerated dose by the historical i3 + 3 trial design (Hi3 + 3) – up to ED_50_ receptor occupancy model and efficacy on pharmacodynamic biomarker assessment: minimal anticipated biological effect level = 7.0 × 10^–9^/kg, active treatment dose = 5.4 × 10^–9^/kg and no observed adverse effect level > 50.7 × 10^–14^ mol/(L·kg) [37] (Additional file 2: Pharmacokinetics of 10 mg TCS10 i.v. injection). The TCS10’s investigational medicinal product critical process parameter was performed by quality assurance by BCN Peptides S.A. Pol.Ind. Els Vinyets-Els Fogars, Barcelona, Spain and Alfa Wassermann SpA, Bologna, Italy, for regulatory Italian Medicines Agency.

### Study objectives

The intention-to-treat (ITT) in primary analysis was to investigate the effect of TCS10 on levels of pro-inflammatory cytokines over time, and its association with O/E prior-risk log-odds of conjugate mortality rate [38]. The second objective aimed to analyze associations between the allocation arm (TCS10 group or control group), the incidence of bleeding episodes and the need for blood transfusion over time.

This analysis took into consideration various factors, including the impact of lactate levels in the bloodstream and the return of time-weighted values for different families of cytokines: tumor necrosis factors (TNF-α and TNF-β), interleukins (IL-6 and IL-10), interferon gamma (IFN-γ), vascular endothelial growth factor (VEGF), Fms-like tyrosine kinase 3 ligand (Flt3L), fractalkine, platelet derived growth factor (PDGF), selectin, serum intercellular cell adhesion molecule 1 (sICAM1), and soluble vascular cell adhesion molecule 1 (sVCAM1).

To collect data at different time points, the following sampling schedule was followed: baseline measurement taken at T_0_, during surgery with a 10-min interval after T_0_ recorded as T_1_, another 20-min interval after T_1_ marked as T_2_, and a final measurement at 18-h after T_2_ denoted as T_3_. This approach allowed for a comprehensive evaluation of the relevant factors over time and their potential influence on primary outcome measures.

### Outcome measures

All cytokines were measured by plasma isolated from peripheral blood samples taken from patients at T_0_, T_1_, T_2_ and T_3_ and transported on ice and immediately centrifuged at 1500 × *g* for 10 min at 4 ℃, and supernatants were aspirated and separated into 500 μl aliquots (Accelera Srl, Aviano, Italy for transport and Levi-Montalcini IRET Foundation High-Tech Labs Bologna, Italy). After collection, serum samples were stored at −80 ℃. Any samples suspected of hemolysis were excluded from this study.

Plasma was assayed for the 13 cytokines by Luminex xMAP immunoassay (Millipore, Billerica, MA, USA). Raw data analysis (mean fluorescence intensity) was performed using a standard five-parameter logistic curve fit created by the Luminex xPONENT^®^ Software (version 3.1). The analysis of plasma lactate was carried out by the commercial kit supplied by Randox (UK) on a semiautomated system 5010 using standards and quality control serum.

### Bleeding-blood bag unit transfusion

The combined primary endpoint was progression free survival and blood transfusion bleeding control. Data on the frequency of bleeding episodes and need for blood transfusion were collected over an 18-h timeframe. The post randomization protocol was based on transfusion strategy of European Society of Intensive Care Medicine as an algorithm of unit of blood for decision making for serial event of bleeding (timing T_0_ to T_3_) [7]. According to this algorithm, the blood unit required is to maintain an average value not less than 7.5–8.0 g/dl (5.0–6.7 mmol/L) of haemoglobin within the first 18*-*h [7].

### Mortality and sensitivity analysis

Survival analysis for all-cause mortality was carried out by the Kaplan-Meier plot and curves (TCS10 group vs. control group) were compared using the log-rank test. Density-based mortality sensitivity index was performed using the prior-risk log-odds prediction information of calibrated and discriminated ES2 Z-score based on systematic review and Meta-analysis, as continuous random variable to posterior distribution in standardized mortality ratio [39, 40]. The likelihood function of calibrated ES2 was used in a tipping point approach in a “reverse-Bayes” method by assessing efficacy in retrospective of critical threshold between prior weight and maximum a posterior of ES2 proportional hazard weight [41]. Details Bayesian sensitivity analysis see Additional file 2: Bayesian sensitivity analysis.

### Statistical analysis

Continuous variables are expressed as mean ± standard deviation (SD) and skewed data are presented as *M* (*Q*_1_, *Q*_3_). The Mann-Whitney *U* test was used to compare patients’ characteristics between the two groups. Biomarkers that showed low degree of variability over time were not included in longitudinal analysis.

The ES2 prior-risk log-odds in parametric were computed by Mann-Whitney *U* of two-sample *U*-statistic likelihood estimation of calibrated Z-AUC of O/E mortality rate according to all data derived from Meta-analysis studies [42]. Bootstrap of predictive factor as Berger-prior/normal log-odds, were adjusted to probability density function (PDF) by macros distribution of β shape *K* factor parameter of characteristic life (η) in conditional Weibull probability distribution survival table [43]. The sensitivity index (equal-variance Gaussian distribution) between hit rate and linear unit β slope macros was considered as equal/unequal variance between samples in any point to the right. The blood-bag-unit transfusion for bleeding episode algorithm was cross validated by the *K*-mean hierarchical clustering on percentage [44].

Linear mixed models (LMMs) were used to compare changes in the levels of cytokines over time between the treatment arms. To meet the assumptions of these models, positively skewed biomarkers were log-transformed before testing. The missing at random (MAR) assumption was examined by testing whether missing values of each biomarker were independent of the observed values.

A Bonferroni correction was applied to adjust for multiple testing. The LMMs (having the repeated measurements of a specific biomarker over time as dependent variable) included the allocation arm, time, and treatment × time interaction term, always adjusting for ES2. If the time × treatment interaction term was not significant, it was removed from the analysis, and the main effect of the allocation arm on the biomarker was examined with time as a covariate.

Logistic regression analysis was used to assess the efficacy of the allocation arm on bleeding and blood transfusion, as well as the influence of baseline variables on the between-arm differences. Univariate and multivariate logistic regression analyses were conducted to evaluate the associations between changes in levels of cytokines and bleeding or transfusion.

Survival analysis was performed using Kaplan-Meier curves and compared using the log-rank test. A *P*-value ≤ 0.05 was considered statistically significant.

All calculations were performed using SPSS (SPSS for Windows version 22, Chicago, IL, USA), Stata software (Stata for Windows version 13, College Station, TX, USA) and R version 3.6.3 (the R Foundation for Statistical Computing, Vienna, Austria).

## Results

### Patient characteristics

A total of 100 patients were enrolled in this study. Apart from a lower frequency of tearing/ripping chest pain reported in TCS10 group compared with control group (48.2% vs. 75.0%, *P* = 0.01), no other significant differences were observed for baseline demographic and clinical characteristics between TCS10 group and control group (*P* > 0.05). The study population included 34 (60.7%) males and 22 (39.3%) females in TCS10 group, 19 (43.2%) males and 25 (56.8%) females in control group. Mean age was (74.4 ± 8.3) years in TCS10 group and (73.2 ± 8.6) years in control group. ES2 score was 12.1 ± 2.7 [logistic (25.6 ± 8.9)%] in TCS10 group and 11.3 ± 1.3 [logistic (22.6 ± 6.3)%] in control group (Table 1).

**Table 1:** Demographic and clinical characteristics of two groups of patients

**Items**	**TCS10 group (*n* = 56)**	**Control group (*n* = 44)**	***P*-value**
Demographic			
Age (years, mean ± SD)	74.4 ± 8.3	73.2 ± 8.6	0.42
Height (cm, mean ± SD)	167.4 ± 9.6	165.73 ± 9.9	0.37
Weight (cm, mean ± SD)	75.6 ± 13.6	72.2 ± 13.9	0.17
BMI (kg/m^2^, mean ± SD)	26.9 ± 4.0	26.5 ± 4.6	0.48
Male/female (*n*)	34/22	19/25	0.08
ES2 score (mean ± SD)	12.1 ± 2.7	11.3 ± 1.3	0.21
History [*n*(%)]			
Out-in hospital admission			
Emergency, critically ill	39(69.6)	33(75.0)	0.56
Thoracic aortic aneurysm	17(30.4)	10(22.7)	0.39
Previous cardiac surgery	11(19.6)	9(20.5)	0.92
In-hospital iatrogenic			
Emergency/rescue	17(30.4)	11(25.0)	0.55
Previous cardiac surgery	10(17.9)	7(15.9)	0.80
Presenting symptoms and signs (pre-surgery out-in hospital) [*n*(%)]			
Respiratory rate > 24 times/min	56(100.0)	44(100.0)	1.00
PaO_2_/FiO_2_ < 90 mmHg	56(100.0)	44(100.0)	1.00
Systolic blood pressure < 90 mmHg	56(100.0)	44(100.0)	1.00
Pulse < 130 min	30(53.6)	25(56.8)	0.75
Temperature < 35 °C	15(26.8)	9(20.5)	0.48
Severe or worst-ever pain for call^a^	18(32.1)	22(50.0)	0.07
Chest pain: anterior for call	15(26.8)	13(29.5)	0.76
Tearing or ripping pain for call^a^	27(48.2)	33(75.0)	0.01
Syncope^a^	2(3.6)	3(6.8)	0.46
Severe hypotension < 60 mmHg^a^	12(21.4)	13(29.5)	0.35
Not induced coma	3(5.4)	1(2.3)	0.43
Normal chest X-ray	4(7.1)	2(4.5)	0.59
Widened mediastinum on chest X-ray	22(39.3)	19(43.2)	0.69
Hemoglobin < 10 g/dl (6.2 mmol/l)	42(75.0)	31(70.5)	0.61
Thromboelastogram LY30 > 10%	15(26.8)	10(22.7)	0.64
ALS (pre-surgery out-in hospital)			
Cardiac arrest [*n*(%)]	10(17.9)	9(20.5)	0.74
Shockable cardiac arrest [*n*(%)]	5(8.9)	3(6.8)	0.70
Asystole time^a^ (min, mean ± SD)	12.4 ± 8.7	11.5 ± 8.6	0.61
CPR time^a^ (min)	40	30	-
Acute respiratory failure [*n*(%)]	56(100.0)	44(100.0)	1.00
Control ongoing bleeding [*n*(%)]	15(26.8)	10(22.7)	0.64
Drugs & fluid^b^ [*n*(%)]			
Adrenaline bolus	56(100.0)	44(100.0)	1.00
High flow oxygen	56(100.0)	44(100.0)	1.00
Initial fluid challenges	56(100.0)	44(100.0)	1.00
Atropine	15(26.8)	10(22.7)	0.64
Calcium chloride	56(100.0)	44(100.0)	1.00
Amiodarone	15(26.8)	10(22.7)	0.64
Tranexamic acid	15(26.8)	10(22.7)	0.64
Maneuver [*n*(%)]			
Chest compression	10(17.9)	9(20.5)	0.74
IABP	7(12.5)	10(22.7)	0.18
Emergency thoracotomy	10(17.9)	9(20.5)	0.74

*ALS* advance life support, *BMI* body mass index, *CPR* cardiopulmonary resuscitation, *ES2* European System for Cardiac Operative Risk Evaluation, *IABP* intra-aortic balloon counterpulsation pump, *PaO*_*2*_*/FiO*_*2*_ partial pressure of oxygen in arterial blood (PaO_2_) to the fraction of inspiratory oxygen concentration (FiO_2_)

^a^Nearest most reliable

^b^ALS Eu-Resuscitation Council Guideline

### Baseline levels of biomarkers

Levels of biomarkers at enrollment (T_0_) are presented in Additional file 1: Table S1. No statistically significant differences were found between TCS10 group and control group for the different cytokines analyzed and lactate, except for IL-6, that was significantly lower in TCS10 group compared with that in control group [0.6 (0.6, 2.8) pg/ml vs. 1.5 (0.6, 9.8) pg/ml, *P* = 0.03].

### Variation in biomarker levels over time

Variations in the levels of biomarkers from T_1_ to T_3_ are shown in Additional file 1: Table S2. To investigate the between-group differences of biomarker levels over time, LMMs of various complexity having each biomarker as dependent variable were fitted. LMMs included the allocation arm as well as ES2 score and time (model 0). These analyses revealed no between group-differences in the evolution over time for the following biomarkers: IL-6 (*P* = 0.08), IL-10 (*P* = 0.73), Flt3L (*P* = 0.47), selectin (*P* = 0.47), sICAM1 (*P* = 0.95) and sVCAM1 (*P* = 0.45) (Additional file 1: Fig. S2, Table S3).

**Fig. 1:**
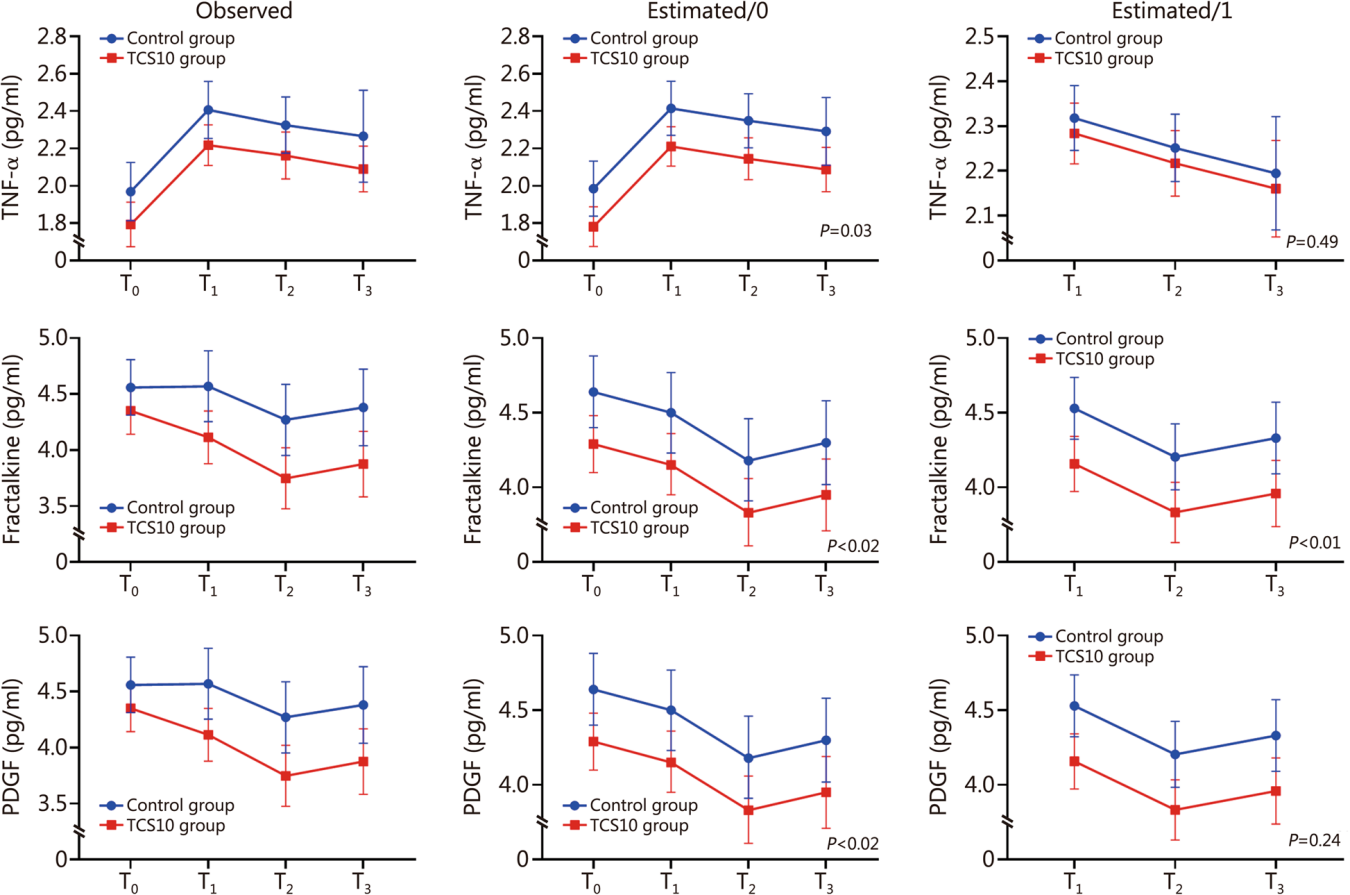
Linear mixed models (LMMs) to evaluate the between-arms differences in TNF-α, fracktalkine and PDGF levels over time in TCS10 group and control group patients at different time points. Data are presented as mean and error bars represent standard deviation. LMMs included the allocation arm as well as ES2 score and time (model 0; estimated/0) and baseline values of these cytokines (model 1; estimated/1). T_0_ baseline, T_1_ 10 min after baseline, T_2_ 20 min after T_1_, T_3_ 18 h after T_2_, TNF tumor necrosis factor, PDGF platelet derived growth factor, ES2 European System for Cardiac Operative Risk Evaluation

**Fig. 2:**
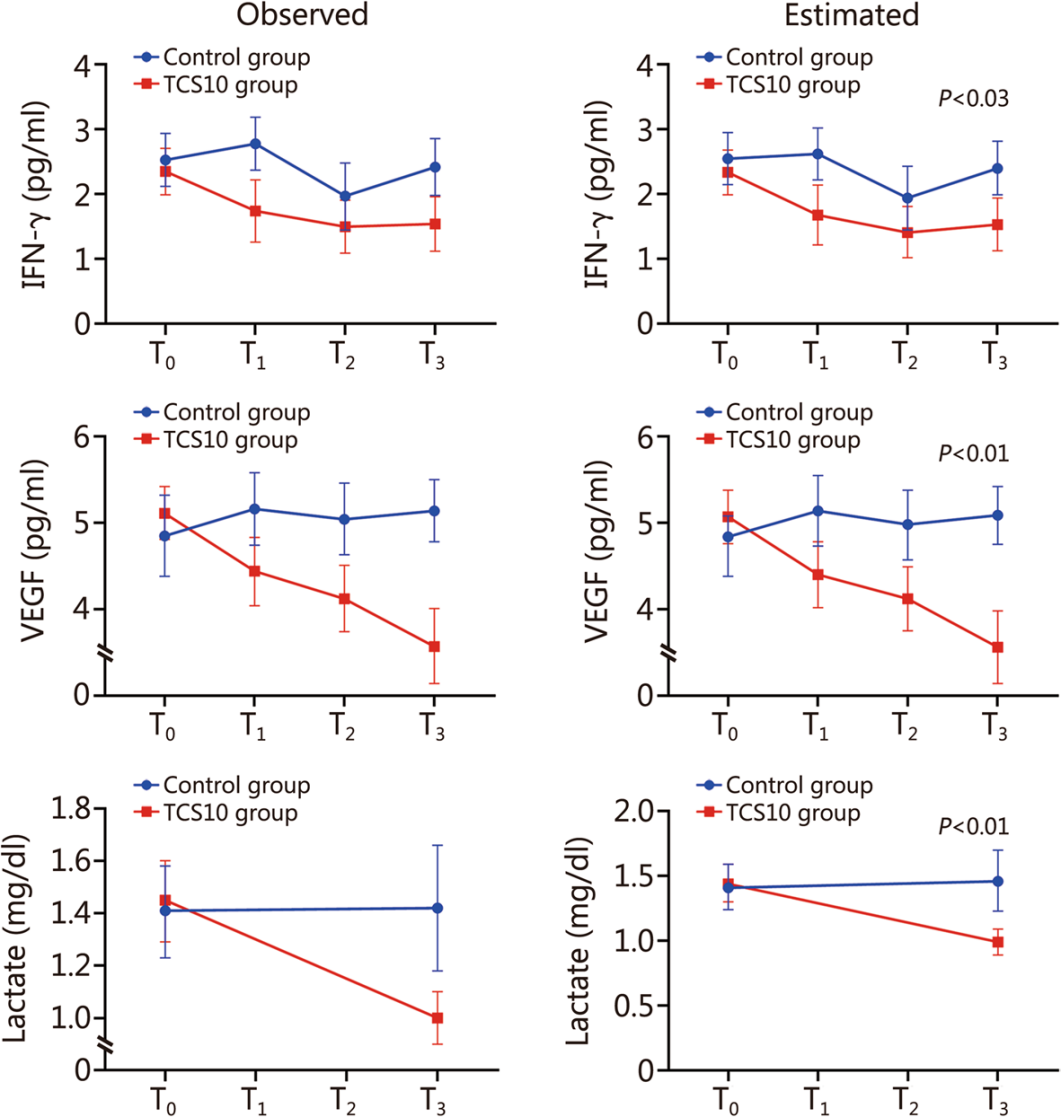
Linear mixed models (LMMs) to evaluate the between-arms differences in IFN-γ, VEGF and lactate levels over time in treated (TCS10 group) and untreated (control group) patients at different time points. Data are presented as mean and error bars represent standard deviation. LMMs included the allocation arm, time, allocation × time interaction term, and ES2 score (model 2). T_0_ baseline, T_1_ 10 min after baseline, T_2_ 20 min after T_1_, T_3_ 18 h after T_2_, IFN-γ interferon gamma, VEGF vascular endothelial growth factor, TNF tumor necrosis factor, ES2 European System for Cardiac Operative Risk Evaluation

When investigating the between-group differences of TNF-α, fractalkine and PDGF over time using model 0, we found that the TCS10 treatment vs. the control treatment induced an advantage at T_1_, which remained constant across time (*P* = 0.03 for TNF-α and *P* = 0.02 for fracktalkine and PDGF; Additional file 1: Tables S2 and S3). Nevertheless, we noted a “carrier over effect” due to the between group-differences in circulating levels of these biomarkers (TNF-α, fracktalkine and PDGF) at baseline which maintained across the study period (model 0; “estimated”; Fig. 1). This baseline imbalance generated an apparent significant effect of the allocation arm across the study period. To account for this imbalance, baseline values of TNF-α, fractalkine and PDGF were introduced as covariates into the corresponding model of each biomarker (model 1, Additional file 1: Table S4). After this adjustment, the effect of allocation arm on TNF-α and PDGF disappeared, whereas that of fractalkine did not (*P* < 0.01, Fig. 1). For IFN-γ, VEGF, and lactate, a significant allocation arm × time interaction was observed. The corresponding LMMs included the allocation arm, time, allocation × time interaction term, and ES2 score (model 2) are presented in Fig. 2. In this model, an advantage at T_1_ in TCS10 group (compared with control group) was observed, which amplified over time (Additional file 1: Table S5). However, at T_1_, a violation of the MAR assumption was found for IFN-γ (Additional file 1: Table S6).

### Association between biomarker levels and frequency of bleeding

Across the follow-up period, 54 patients experienced bleeding episodes. Among these, 27 patients were observed in TCS10 group (27/56 = 0.48) and the remaining 27 in control group (27/44 = 0.61). The difference [reduction in odds ratio (*OR*): -41%] failed to reach statistical significance [*OR* = 0.59, 95%CI 0.26–1.31, *P* = 0.19]. Therefore, the allocation arm did not significantly affect the incidence of bleeding, although the magnitude of the reduction was clinically relevant. Although we did not observe a significant effect of the allocation arm on bleeding episodes, the number needed to treat (NNT) was considered very satisfactory, resulting in 8 [1/(0.61–0.48) =  ~8]. This implies that for every 8 patients treated with TCS10, 1 bleeding case is avoided.

Beyond the main effect of the allocation arm on bleeding episodes, we also investigated whether some variables assessed at baseline modified the efficacy of the experimental intervention on bleeding. This analysis revealed that the effect of TCS10 treatment on bleeding had no significant impact on patients’ strata (including ES2 score), indicating that no effect modification by baseline variables influenced the allocation arm-bleeding link (*P*-value ranging from 0.13 to 0.81, Additional file 1: Table S7).

Univariate associations between log transformed baseline values of each biomarker and bleeding are summarized in Table 2. Results reveal that IL-6, IL-10 and lactate are the only cytokines directly associated with bleeding, while no effect of drug administration on bleeding episodes was found (Additional file 1: Table S8). In contrast to results presented in Additional file 1: Table S7, we performed additional analyses to assess the impact of Flt3L as a continuous log-transformed variable, rather than using its absolute median value. Our findings revealed a significant modification effect of Flt3L on the association between treatment and bleeding (*P* = 0.03, Additional file 1: Table S9). Specifically, we observed that higher levels of Flt3L were associated with lower *OR* for bleeding episodes, indicating a greater effectiveness of the treatment. The effect modification of Flt3L at predefined biomarker levels corresponding to quartiles was calculated. In the 3rd quartile of Flt3L, the treatment exhibited a pronounced (69%) reduction in the *OR* of bleeding episodes (*OR* = 0.31, 95%CI 0.11–0.90, *P* = 0.03; Additional file 1: Table S9).

**Table 2:** Univariate logistic regression of presence/absence of bleeding (dependent variable) according to baseline biomarkers (independent variables)

**Log (cytokine; pg/ml)**	***OR***^**a**^	**95%CI**	***P*-value**
Log (TNF-α)	1.95	0.85–4.69	0.12
Log (TNF-β)	0.98	0.53–1.89	0.96
Log (IL-6)	1.90	1.39–2.79	0.00
Log (IL-10)	1.53	1.17–2.12	0.00
Log (IFN-γ)	0.84	0.61–1.13	0.25
Log (VEGF)	0.84	0.60–1.14	0.29
Log (Flt3L)	0.93	0.73–1.18	0.56
Log (fractalkine)	0.85	0.50–1.40	0.52
Log (PDGF)	0.87	0.64–1.13	0.34
Log (selectin)	1.35	0.63–2.97	0.44
Log (sICAM1)	0.92	0.36–2.33	0.85
Log (sVCAM1)	0.57	0.13–2.37	0.44
Log (lactate)	2.92	1.40–6.66	0.01

*Flt3L* Fms-like tyrosine kinase 3 ligand, *IFN-γ* interferon gamma, *IL* interleukin, *sICAM1* serum intercellular cell adhesion molecule 1, *sVCAM1* serum vascular cell adhesion molecule 1, *TNF* tumor necrosis factor, *PDGF* platelet derived growth factor, *VEGF* vascular endothelial growth factor

^a^Refers to 1-unit log (natural logarithm) increase

### Association between blood transfusion and cytokine levels

Forty-nine patients were transfused with > 3 blood units, 50 patients with < 3 blood units, and 1 patient had missing information regarding transfusion. The *OR* of transfusion was significantly lower in TCS10 group (21/56 = 0.38) than that in control group (28/43 = 0.65, for 1 patient, there was missing information regarding transfusion) (*OR* = 0.32, 95%CI 0.14–0.73, *P* = 0.01). The NNT was very satisfactory, resulting in ~ 4 [1/(0.65–0.38) =  ~4]. This implies that for every 4 patients treated with the experimental drug, 1 transfusion case is avoided.

The analysis reported in Table 3 revealed that the effect of TCS10 treatment had no impact on patients’ strata (including ES2 score) indicating that no effect modification by baseline variables influenced the allocation arm-transfusion link (*P*-values ranging from 0.13 to 0.87). No significant effect of baseline cytokines levels on blood units transfused was found (Additional file 1: Table S10). Adjustment for baseline levels did not affect the treatment-transfusion link (Additional file 1: Table S11).

**Table 3:** Logistic regression analyses of presence/absence of transfusion (dependent variable) according to the allocation arm (independent variable) and stratified by relevant patients’ strata

**Potential effect modifier**	***OR*(95%CI) (TCS10 vs. control)**	***P*-value (TCS10 vs. control)**	***P*-value for effect modification**
Age (years)			0.58
Median (75.5)	0.41 (0.14–1.25)	0.13	
Median (75.5)	0.26 (0.02–3.30)	0.30	
Gender			0.26
Female	0.19 (0.05–0.65)	0.01	
Male	0.52 (0.16–1.57)		
BMI (kg/m^2^)			0.13
Median (26.1)	0.15 (0.04–0.53)	0.01	
Median (26.1)	0.58 (0.18–1.86)	0.36	
ES2 score			0.31
Median (20.5)	0.21 (0.06–0.69)	0.01	
Median (20.5)	0.50 (0.16–1.59)	0.24	
TNF-α (pg/ml)			0.56
Median (6.6)	0.41 (0.13–1.34)	0.14	
Median (6.6)	0.25 (0.08–0.83)	0.02	
IL-6 (pg/ml)			0.44
Median (0.9)	0.27 (0.08–0.90)	0.04	
Median (0.9)	0.53 (0.16–1.76)	0.30	
IL-10 (pg/ml)			0.50
Median (0.6)	0.24 (0.07–0.77)	0.02	
Median (0.6)	0.46 (0.12–1.75)	0.25	
IFN-γ (pg/ml)			0.55
Median (12.2)	0.24 (0.27–0.81)	0.02	
Median (12.2)	0.40 (0.13–1.29)	0.13	
VEGF (pg/ml)			0.44
Median (183.3)	0.23 (0.07–0.79)	0.02	
Median (183.3)	0.46 (0.14–1.54)	0.21	
Flt3L (pg/ml)			0.58
Median (9.1)	0.27 (0.08–0.92)	0.04	
Median (9.1)	0.43 (0.13–1.43)	0.17	
Fractalkine (pg/ml)			0.37
Median (91.1)	0.23 (0.07–0.77)	0.02	
Median (91.1)	0.49 (0.15–1.56)	0.23	
PDGF (pg/ml)			0.45
Median (2940)	0.44 (0.14–1.37)	0.16	
Median (2940)	0.23 (0.06–0.82)	0.02	
Selectin (pg/ml)			0.24
Median (63.3)	0.46 (0.14–1.46)	0.19	
Median (63.3)	0.15 (0.04–0.64)	0.01	
sICAM1 (pg/ml)			0.40
Median (69.5)	0.43 (0.13–1.39)	0.16	
Median (69.5)	0.20 (0.05–0.76)	0.02	
sVCAM1 (pg/ml)			0.87
Median (783.5)	0.37 (0.11–1.20)	0.10	
Median (783.5)	0.32 (0.10–1.04)	0.06	
Lactate (mg/dl)			0.84
Median (3.9)	0.35 (0.11–1.10)	0.07	
Median (3.9)	0.29 (0.09–0.97)	0.05	

*BMI* body mass index, *Flt3L* Fms-like tyrosine kinase 3 ligand, *IFN-γ* interferon gamma, *IL* Interleukin, *sICAM1* serum intercellular cell adhesion molecule-1, *sVCAM1* serum vascular cell adhesion molecule-1, *TNF* tumor necrosis factor, *PDGF* platelet derived growth factor, *VEGF* vascular endothelial growth factor

### Survival analysis

Kaplan-Meier survival analysis showed that all death cases (*n* = 8, 7 for shock and 1 for multi-organ failure) occurred in control group whereas no deaths were observed in TCS10 group. One death occurred in the first 24 h. The last case of death was observed after 408 h (17 d). The two survival curves significantly differed between groups (log-rank *P* < 0.001, Additional file 1: Fig. S3).

### Bayesian sensitivity analysis

The Meta-analytic-predictive prior informative generation for comparison was discriminated by ES2 score *U*-statistic log-odds resulted in an *OR* = 1.12 (95%CI 0.94–1.29) [pooled Meta-analytic, sample size 132,516, as R-squared death rates and probability distribution in Gaussian-based ROC curve for a rating coefficient (Z-AUC)]. The calibrated O/E for both groups resulted in sensitivity of 75.0% and specificity of 77.2% (Z-AUC = 0.78, 95%CI 0.59–0.78, *P* = 0.0007), the observed mortality rate of 18% in control group had a sensitivity of 75.0% and specificity of 80.6% (Z-AUC = 0.79, 95%CI 0.64–0.89, *P* = 0.0003).

The TCS10 treated resulted in an observed mortality calibration of any threshold equal to 0 with significant difference for control grouping Z-AUC, O/E area (95%CI 0.21–0.70, *P* = 0.0002).

The lactate clearance thresholds for both groups of 37%, 30-day mortality (≥ 64% Δ24-h) crosslinking to Z-AUC expected mortality, resulted in a sensitivity of 41.20% and specificity of 96.30% (Z-AUC = 0.69, 95%CI 0.53–0.82, *P* = 0.034). The control group prediction resulted in a sensitivity of 88.24% and specificity of 96.30% (Z-AUC = 0.96, 95%CI 0.86–0.99, *P* < 0.0001, associated criterion > 4.7). The TCS10 treated group resulted in 33.9% for prediction positive cases comparing the accuracy calibration to observed mortality of 0 at any threshold.

The cumulative credit rating *R*-coefficient weight to death rates and probability distribution resulted respectively in control group of 0.83 for ES2 and 1.14 for lactate clearance thresholds respect to TCS10-treated patients of 0.00 for both ES2 and lactate.

The log-rank (Mantel-Cox) were within a *χ*^2^ = 11.1, *P* = 0.001. In log-rank test, *P* < 0.001 truncated time at 600 h survival.

The bootstrap of ES2 Z-score in PDF distribution of Weibull shape parameter of regression parametric regression model was for macros *K*-factor at left of about 1.47 in control group and about 1.31 in TCS10 group, respectively to right of 1.70 for control and 8.90 for TCS10-treated with ratio between them distribution assessment in last square parameter of 0.19 and in calibrated O/E of 1.15 for control vs. 6.80 of TCS10-treated.

The relative risk ratio (*RRR*) for 30-day mortality rate was 0.046 (one-sided: 95%CI 0.0027–0.78, *P* = 0.03; *RRR* = 0.900; two-sided: 95%CI 0.21–3.80) equating to an 18-fold decrease in mortality risk by treatment (*P* = 0.03). The *χ*^2^ (trend) was significant (*P* = 0.0034) in TCS10 group for bleeding episodes (*P* = 0.0049) and for blood-bag-unit transfusion performed at 24 h (*P* = 0.0085).

The NNT was estimated at 5.6 for the number of patients who need to be treated with the new treatment rather than the standard treatment for one additional patient to benefit. 95%CI is 3.5–13.4 (benefit; *P* = 0.03) which is sensitive for avoiding one bag of blood unit for every 4 (25.0%) and 1 (12.5%) bleeding that exceeds 8 [45].

## Discussion

Sensitivity analysis plays a crucial role in assessing the robustness of the findings or conclusions based on primary analyses of data derived from clinical trials [46]. This dimension is particularly useful for our goal in understanding RCT’s explanatory context when reviewing datasets from left to right of hidden multiple imputation of immunity ranks when censoring time-to-event [47]. The ITT identified the hardness of melanocortin targeting receptor in a two-spin operator for driving the cAMP-dependent pathway within the temporal window of criticality of host response as certainly state-condition and unique topology closed to the uncertainty of diagnosis and care. Semantically, it is more appropriate to define these types of patients as a state of probability of death due to the host’s uncertain response to its own terminal spatial-temporal phenomenology [48]. The rationale for treatment is to achieve local stability of binding energy and molecular docking across the supply chain in a time-to-event equilibrium. The objective is to contrast the hyperinflammation càdlàg forces in scalar values for rapid structural transformation and bearing capacity in the microcirculatory system.

The Emergency Medical Care (EMC) scenario was projected in competing risk event happens in situations where multiple events are likely to occur in a way that the occurrence of one event may prevent other events from being observed in risk assessment. The RCT’s hazard distribution is an assumption by ES2 to rely on normal distribution for continuous outcomes, Poisson distribution for count data in binomial distribution for binary outcome data. Thus, outcome proportional hazard is based on the likelihood rule of Bayesian Nash and the Cauchy-Euler equation of uncertainty [49]. The Emerging Bayesian’s Contingent Operator as counterpart is hyperinflammation that can be defined as an angular momentum of immune metabolism [49]. In this regard, the RCT followed the immunometabolism direction of interdependence from left to right as Bayesian operator for structural dynamics and duopoly constraint function in a group based on the common threshold of 30-day fatality rate [25].

The test-retest logarithm confidence level concerns independent stratum variables within ES2 likelihood function are metric of area under ROC and logarithmic scale as calculated Z-score associate with AUC sensitivity and specificity in screened O/E probability coefficient. Phase 3 is the mainstay of alpha (1–24)-corticotrophin in TCS10 drug development across a wide range of scientific studies that have often met expectations clinically significant and consistently with higher survival rates in animal and human studies prior to the publication of RCTs [50–52].

The increased knowledge gained from RCTs was attributed to new discoveries in bioinformatics and immunometabolism, which led to new nomenclature and classification of any time-space operator pharmacokinetic studies. Results derived from the primary analysis of this study revealed a progressive free survival of 255 d for all patients who received TCS10, compared with 17 d or less for 8 patients in the control group. The cross-survival factors estimated the net benefit for bleeding control and blood bags transfusion units’ consumption. The biomarker was significant in lactate clearance and 4 classes of cytokine expression. However, in primary analysis a low number of patients who died (*n* = 8, all recorded in the control group of the trial), precludes the possibility to investigate the effect of modification by biomarkers on the relationship between the allocation arm and mortality. In fact, according to the rule of thumb of 1 variable into the model for every 10 patients with the event of interest, such an effect modification analysis (3 parameters requiring at least 30 deaths cases instead of 8 (i.e., those observed).

The lack of information on the time of occurrence of bleeding episodes did not allow the possibility to evaluate the temporal change in biomarkers changes and the occurrence of bleeding episodes (i.e., cox-regression analysis). To address this issue, survival curve analysis using the Kaplan-Meier estimator was re-calibrated in weighted probability in “reference skip” in a reference-based imputation of multi-state observation.

The Bayesian paradigm and Gibbs sampler share the probabilities directly from the self-tracking line of the structural function in an accelerated failure as a result. In practice, the observation of many complex systems exhibits thresholds involving an abrupt and irreversible change in a dynamical regime. Such events are commonly referred to as a “goodness-of-fit” for sensitivity by the symmetric, bell-shaped distribution of Student-T distribution for count data of negative-binomial distribution to over-dispersion the PDF. The Stochastic random walk is the intersection of metabolic pathways, intermediaries up to mitochondrial dynamics and mitophagies marker to regulate the functions of two-way immune cells. Typically, the critical unidirectional loop over time, interacts with the temporal order of endothelial surface function and the cohesive response of the hematological system as failure and malfunction [53].

This combination of life-threatening state for incoming circulatory failure causes inadequate oxygen delivery to meet cellular metabolic needs and oxygen consumption requirements, producing cellular and tissue hypoxia. The initial reversibility is known, but it can quickly become irreversible, leading to late multiorgan failure and death. Current research seeks to develop early warning for “critical” that could help and prevent undesirable events such as collapse. Clinically, it is essential that the stratification of severity and prognosis as a homogeneous group classification requires intervention without delay and is included in prediction and algorithms that bring together the global rule of vital signs. The meta-analysis indicated that few independent variables are considered the existing medical condition as terminal illness for premature death. The immunity rank is for adaptive interface as a space zone operator of this phenomenology [54].

Reanalysis demonstrates a significant reduction in the requirement for blood transfusion in the TCS10 group compared to the control group (*OR* = 0.32, 95%CI 0.14–0.73, *P* = 0.01). Clinically, we observed that by using TCS10, one transfusion case could be avoided for every four. It is important to note that no similar studies have been conducted on the effects of melanocortin’s targeting receptor as TCS10 in criticality and impact on transfusion avoidance. Several markers have been suggested for monitoring multiorgan failure and prognosis of hemorrhagic shock.

Considering the complexity of the condition, which involves numerous cellular and organ targets, in this RCT, attention was paid in particular to cytokines (i.e., IL-6, IL-10, TNF-α, TNF-β, and IFN-γ), markers of endothelial dysfunction (i.e., selectin, sICAM, and sVCAM), cardiovascular markers (i.e. PDGF, VEGF, and Flt3L), and “surrogate markers” for adhesion proteins, as fractalkine. Among these biomarkers, we observed that TCS10 treatment induced a decrease in serum concentrations of fractalkine and VEGF. This reduction remained constant or increased over time for fractalkine and VEGF, respectively. To our knowledge, no previous studies have examined the impact of TCS10 on the temporal changes in fractalkine and VEGF in critically ill patients.

Fractalkine, also known as CX3CL1, is synthesized as a transmembrane molecule that is expressed on the endothelial cell surface. As such, the chemokine can interact with its receptor CX3CR1, which is expressed on monocytic cells, T cell subsets, and NK cells [55]. Pro-inflammatory stimuli can induce the expression of CX3CL1, which then promotes adhesion of monocytic cells. Thereafter, the activation of CX3CR1 can induce cell migration of the adherent monocytes leading to transmigration through the endothelial cell layer [56]. In addition to its activity as a transmembrane adhesion molecule, CX3CL1 can act as a classical chemoattractant for leukocytes when it is shed from the cell surface by the activity of a disintegrin and metalloproteinase domain-containing protein-10 (ADAM10) and ADAM17 [57].

VEGF is probably the most well-known angiogenic inducer, whose role has been investigated in both normal and pathological angiogenesis. The levels of circulating VEGF increase in response to hypoxia, inflammation, and immunopathological processes [58]. In this scenario, the efficacy of TCS10 in lowering and maintaining the level of these two cytokines may depend on its non-adrenal-mediated anti-inflammatory effect, that involves, on the one hand the activation of MCRs (predominantly MC1R and MC3R) on immunocytes and endothelial cells, with consequent inhibition of the translocation of the ubiquitous early transcription factor NF-κB into the nucleus and of the ensuing, NF-κB-dictated, overproduction of several factors of the inflammatory response; from the other hand the triggering of the “inflammatory reflex” or “brain cholinergic anti-inflammatory pathway”, which through the activation of α7-nicotinic receptors on immunocytes inhibits the overproduction and release of inflammatory cytokines while stimulating the production and release of anti-inflammatory cytokines.

In this study, in addition to baseline lactate levels (*OR* = 2.92, 95%CI 1.40–6.66) we also found that high baseline serum concentration of IL-6 and IL-10 were directly associated with bleeding (*OR* = 1.90, 95%CI 1.39–2.79; *OR* = 1.53, 95%CI 1.17–2.12, respectively), independently from treatment. IL-6 and IL-10 belong to pro-inflammatory and anti-inflammatory cytokines family, respectively. The acute phase immune response of post-surgical trauma is mainly regulated by the rapid production and release of various endogenous cytokines, that are signaling intercellular substances that initiate, amplify, and perpetuate inflammatory response on a local and systemic site.

The simultaneous production of anti-inflammatory cytokines may potentially counteract pro-inflammatory cytokine effects and modify the intensity of the inflammatory response. The overproduction of either pro-inflammatory cytokines or anti-inflammatory cytokines may result in organ dysfunction [59]. This could explain why both IL-6 and IL-10, although the opposite function in inflammatory pathway, had higher baseline serum concentrations in patients with higher probability of bleeding. In contrast to the findings for IL-6 and IL-10, baseline Flt3L serum concentrations were found to be associated with the response of bleeding after TCS10 treatment. Our study revealed that high serum concentrations of Flt3L resulted in a 79% reduction in the frequency of bleeding episodes in the treated group (*OR* = 0.31, 95%CI 0.11–0.90, *P* = 0.03). Flt3L is a growth factor that plays a role in maintaining and proliferating hematopoietic stem and progenitor cells [60].

Additionally, it has been found that MC5R signal transduction is necessary to induce enucleation [61]. This suggests that Flt3L may synergize with TCS10 in promoting the proliferation of hematopoietic progenitor cells, leading to a reduction in bleeding episodes. Finally, serum lactate concentration (baseline levels were observed to be significantly associated with bleeding; *OR* = 2.92, 95%CI 1.40–6.66) is a well-known independent prognostic marker in ICU and serves as a guide for resuscitation in critically ill patients [62].

While previous studies have demonstrated the beneficial effects of melanocortins (e.g., ACTH, TSC10 and α-MSH) in extreme life-threatening conditions [22–25], the precise mechanisms involved are not yet fully understood. One area of interest to further explore this aspect would be to differentiate between a cortisol spike and melanocortin-driven anti-inflammatory activity and the specific mechanisms underlying each response. In this regard, study has previously demonstrated that melanocortin peptides, such as α-MSH, can exert significant anti-inflammatory effects via activation of MCRs, that are independent of cortisol [63].

Catania et al. [16] demonstrated the anti-inflammatory properties of α-MSH mediated through MC1r activation were independent of cortisol release. Similarly, Colombo et al. [64] showed that α-MSH reduced inflammation in a mouse model of acute lung injury, suggesting cortisol-independent mechanisms. Furthermore, MCR agonists such as BMS-470539 have also shown promising anti-inflammatory effects in preclinical models [65]. In addition, studies involving MC2r-independent agonists such as PT-141 could provide insight into cortisol-independent anti-inflammatory mechanisms [66].

α-MSH primarily targets MC1r, MC3r, MC4r, and MC5r, with minimal affinity for MC2r [67]. Comparing the effects of tetracosactide with α-MSH or other MC2r-independent agonists such as α-MSH or PT-141 in comparative studies may help discern the necessity of cortisol spikes for enhanced anti-inflammatory activity during melanocortin pathway augmentation.

## Conclusion

This sensitivity analysis suggests that a single-dose bolus injection of 10 mg TCS10 holds a first-line option for managing criticality in patients in presence of undifferentiated and incoming shock. Efficacy is for a limited time while transfer for rapidly identifying the etiology and occasional care intervention. The early administration of TCS10 demonstrates beneficial effects on the inflammatory response, resulting in a reduction in the need for blood transfusion and in-hospital improved survival.

These results highlight the potential use of TCS10 both in combat and tactical EMC as a valuable intervention response in the clinical management of pre-hospital emergencies. However, to achieve the technology readiness level in real-life critically ill patients, further large-scale studies are required or Phase IV studies are warranted to validate and expand upon our findings. This will provide a more comprehensive understanding of the efficacy and potential real-life applications in the general population and mass casualty events.

### Supplementary Information

**Additional file 1: Fig. S1** Trial flowchart. **Fig. S2** Linear mixed models (LMMs) to evaluate the between-group differences in IL-6, IL-10, Flt3L, selectin, sICAM1 and sVCAM1 levels over time in TCS10 group and control group patients at different time points. **Fig. S3** Kaplan-Meier survival curve in critically ill patients of TCS10 group and control group. **Table S1** Baseline levels of biomarkers of two groups of patients [*M* (*Q*_1_, *Q*_3_)]. **Table S2** Circulating levels of biomarkers across time from T_1_ to T_3_ [*M* (*Q*_1_, *Q*_3_)]. **Table S3** Bivariate models by natural logarithm of biomarker adjusted by ES2 score. **Table S4** Bivariate models on natural logarithm of TNF-α, PDGF and fractalkine adjusted by ES2 score and cytokine score at T_0_. **Table S5** Bivariate models on natural logarithm of IFN-γ, VEGF and lactate adjusted by ES2 score for treated (TCS10 group) and untreated (control group) patients. **Table S6** Frequency of unavailable data (missing or degraded) according to biomarkers and time points and Mann-Whitney *U *test for testing MAR assumption. **Table S7** Logistic regression analyses of presence/absence of bleeding according to the allocation arm and stratified by relevant patients’ strata. **Table S8** Bivariate logistic regression analyses of baseline biomarkers and treatment on bleeding. **Table S9** Effect modification by baseline Flt3L (log transformed) on the relationship between treatment and bleeding. **Table S10** Univariate logistic regressions by cytokine at baseline on blood units transfused. **Table S11** Bivariate logistic regression analyses of baseline biomarkers and treatment on transfusion.

**Additional file 2.**

## Data Availability

The datasets used and/or analyzed during the current study are available from the corresponding author on reasonable request but restrictions apply to a non-disclosure agreement for National Plan of Military Research, and so are not publicly available.

## Abbreviations

ACTH: Adrenocorticotropic hormone
AUC: Area under receiver operating characteristic;
α-MSH: Alpha-melanocyte-stimulating hormone
cAMP: Cyclic adenosine monophosphate
EMA: European Medicines Agency
EMC: Emergency Medical Care
ES2: European System for Cardiac Operative Risk Evaluation
eCRFs: Electronic case report forms
Flt3L: Fms-like tyrosine kinase 3 ligand
FDA: Food and Drug Administration
ICH: International Council for Harmonization
ICU: Intensive care unit
IFN-γ: Interferon gamma
IL: Interleukin
ITT: Intention-to-treat
IUPHAR/BPS: International Union of Basic and Clinical Pharmacology and British Pharmacological Society
KMI: Kaplan-Maier imputation
LMM: Linea mixed model
MCR: Melanocortin receptor
MAR: Missing at random
NNT: Number needed to treat
NEWS2: National Early Warning Score 2
NF-κB: Nuclear factor kappa-B
O/E: Observed-to-expected
*OR*: Odds ratio
PDF: Probability density function
PDGF: Platelet derived growth factor
RCT: Randomized controlled trial
ROC: Receiver operating characteristic
SOP: Standard operating procedure
sICAM1: Serum intercellular cell adhesion molecule 1
sVCAM1: Soluble vascular cell adhesion molecule 1
TNF: Tumor necrosis factor
VEGF: Vascular endothelial grow factor
Z-AUC: Z-score area under ROC curve
