# Supplementary material for: Effect of early administration of tetracosactide on mortality and host response in critically ill patients requiring rescue surgery: a sensitivity analysis of the STOPSHOCK phase 3 randomized controlled trial
Source: Mil Med Res. 2024 Aug 19;11:56. doi: 10.1186/s40779-024-00555-2 (PMC11331742; doi:10.1186/s40779-024-00555-2)
Supplement: Supplementary file 1 — Additional file 1: Fig. S1 Trial flowchart. Fig. S2 Linear mixed models (LMMs) to evaluate the between-group differences in IL-6, IL-10, Flt3L, selectin, sICAM1 and sVCAM1 levels over time in TCS10 group and control group patients at different time points. Fig. S3 Kaplan-Meier survival curve in critically ill patients of TCS10 group and control group. Table S1 Baseline levels of biomarkers of two groups of patients [M (Q1, Q3)]. Table S2 Circulating levels of biomarkers across time from T1 to T3 [M (Q1, Q3)]. Table S3 Bivariate models by natural logarithm of biomarker adjusted by ES2 score. Table S4 Bivariate models on natural logarithm of TNF-α, PDGF and fractalkine adjusted by ES2 score and cytokine score at T0. Table S5 Bivariate models on natural logarithm of IFN-γ, VEGF and lactate adjusted by ES2 score for treated (TCS10 group) and untreated (control group) patients. Table S6 Frequency of unavailable data (missing or degraded) according to biomarkers and time points and Mann-Whitney U test for testing MAR assumption. Table S7 Logistic regression analyses of presence/absence of bleeding according to the allocation arm and stratified by relevant patients’ strata. Table S8 Bivariate logistic regression analyses of baseline biomarkers and treatment on bleeding. Table S9 Effect modification by baseline Flt3L (log transformed) on the relationship between treatment and bleeding. Table S10 Univariate logistic regressions by cytokine at baseline on blood units transfused. Table S11 Bivariate logistic regression analyses of baseline biomarkers and treatment on transfusion. [file 40779_2024_555_MOESM1_ESM.pdf]

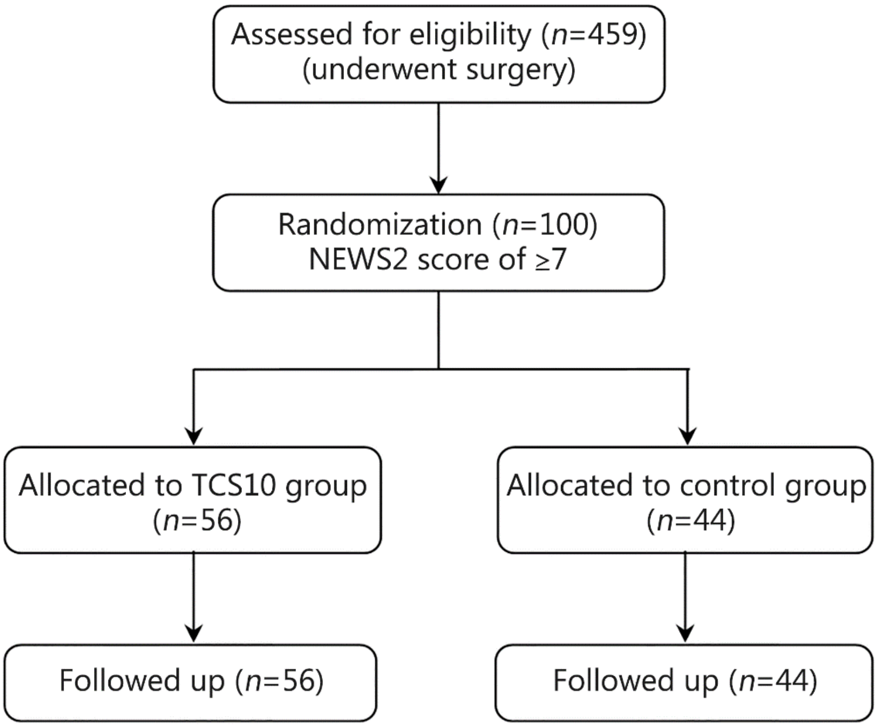

Fig. S1 Trial flowchart. NEWS2 National Early Warning Score 2

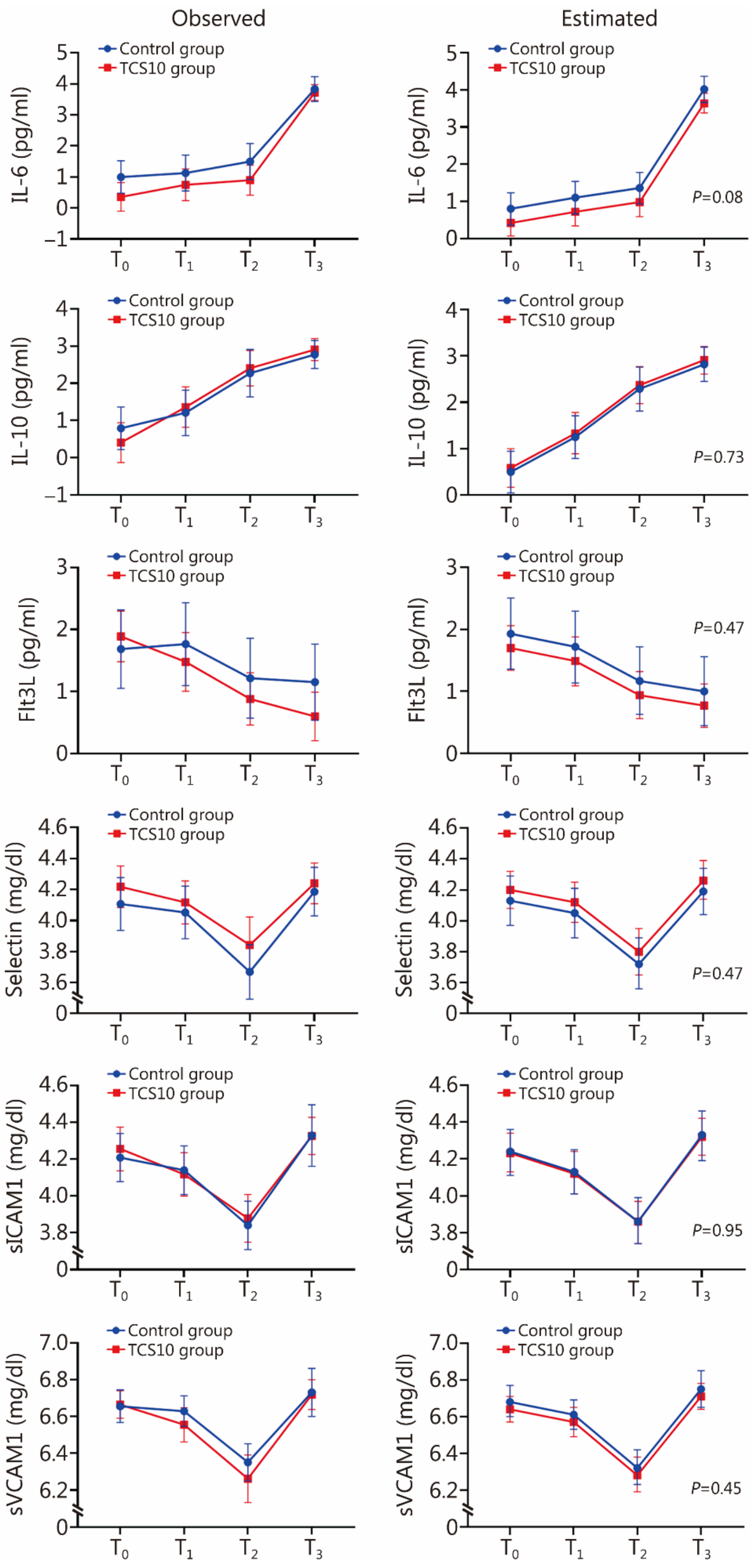

Fig. S2 Linear mixed models (LMMs) to evaluate the between-group differences in IL-6, IL-10, Flt3L, selectin, sICAM1 and sVCAM1 levels over time in TCS10 group and control group patients at different time points. Level of tumor necrosis factor (TNF)-β was not observed to change over time (data not shown). Data are

presented as mean and error bars represent standard deviation. LMMs included the allocation arm as well as ES2 score and time (model 0). T_0 baseline, T_1 10 min after baseline, T_2 20 min after T_1, T_3 18 h after T_2, Flt3L Fms-like tyrosine kinase 3 ligand, IL interleukin, sICAM1 serum intercellular cell adhesion molecule 1, sVCAM1 serum vascular cell adhesion molecule 1, ES2 European System for Cardiac Operative Risk Evaluation

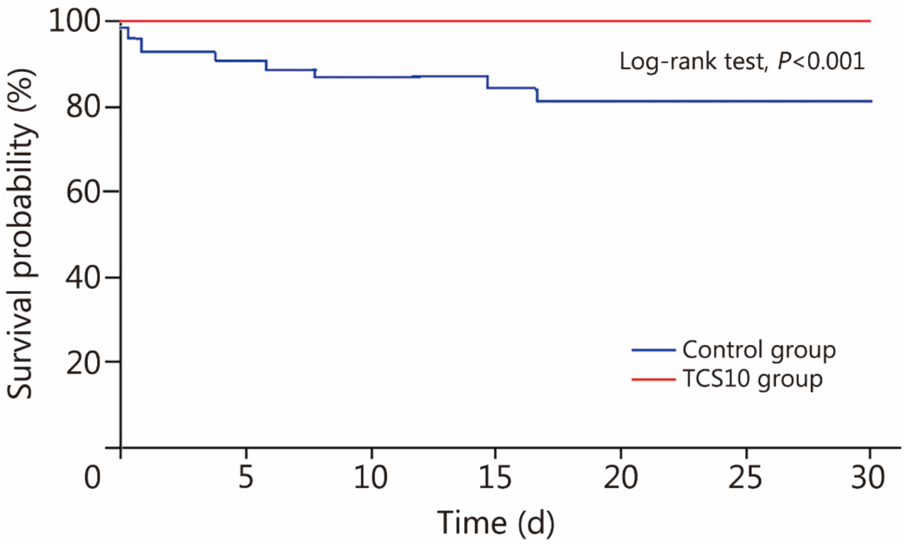

Fig. S3 Kaplan-Meier survival curve in critically ill patients of TCS10 group and control group

Table S1 Baseline levels of biomarkers of two groups of patients [M (Q_1, Q_3)]

**Biomarker**	**TCS10 group (*n* = 56)**	**Control group (*n* = 44)**	***P*-value**
TNF-α (pg/ml)	6.1 (4.4, 7.6)	6.9 (5.3, 10.8)	0.08
TNF-β (pg/ml)	0.6 (0.6, 0.6)	0.6 (0.6, 0.6)	0.14
IL-6 (pg/ml)	0.6 (0.6, 2.8)	1.5 (0.6, 9.8)	0.03
IL-10 (pg/ml)	0.6 (0.6, 1.9)	0.6 (0.7, 7.7)	0.25
IFN-γ (pg/ml)	11.5 (5.5, 23.3)	12.8 (5.0, 29.5)	0.55
VEGF (pg/ml)	203.0 (118.5, 307.2)	176.3 (128.6, 267.9)	0.39
Flt3L (pg/ml)	9.8 (3.2, 20.6)	3.2 (0.6, 31.7)	0.67
Fractalkine (pg/ml)	84.6 (59.4, 118.5)	100 (55.6, 158.6)	0.35
PDGF (pg/ml)	3251.1 (1789.2, 5242.6)	2266.6 (894.4, 5806.9)	0.17
Selectin (pg/ml)	64.5 (51.4, 87.2)	61.8 (42.1, 89.9)	0.57
sICAM1 (pg/ml)	75.3 (55.7, 88.2)	64.1 (52.8, 87.7)	0.49
sVCAM1 (pg/ml)	781.1 (642.8, 958.3)	785.9 (679.5, 903.3)	0.93
Lactate (mg/dl)	3.8 (2.9, 5.3)	3.8 (2.8, 5.2)	0.40

Flt3L Fms-like tyrosine kinase 3 ligand, *IFN-γ* interferon gamma, *IL* interleukin, *PDGF* platelet derived growth factor, *sICAM1* serum intercellular cell adhesion molecule 1, *sVCAM1* serum vascular cell adhesion molecule 1, *TNF* tumor necrosis factor, *VEGF* vascular endothelial growth factor

Table S2 Circulating levels of biomarkers across time from T_1 to T_3 [M (Q_1, Q_3)]

Biomarker	TCS10 group (*n* = 56)			Control group (*n* = 44)		
	T_1	T_2	T_3	T_1	T_2	T_3
TNF-α (pg/ml)	9.3 (7.1, 11.9)	8.6 (6.2, 10.9)	8.0 (5.8, 11.6)	11.3 (8.0, 16.0)	10.8 (6.8, 13.9)	10.0 (6.9, 15.2)
TNF-β (pg/ml)	0.6 (0.6, 0.6)	0.6 (0.6, 0.6)	0.6 (0.6, 0.6)	0.6 (0.6, 0.6)	0.6 (0.6, 0.6)	0.6 (0.6, 0.6)
IL-6 (pg/ml)	1.2 (0.6, 4.6)	1.8 (0.6, 8.0)	40.2 (26.7, 87.2)	2.6 (0.6, 16.3)	3.5 (1.1, 16.3)	49.1 (26.4, 81.4)
IL-10 (pg/ml)	2.2 (0.6, 10.6)	9.3 (3.7, 41.4)	18.3 (9.7, 39.5)	2.3 (0.6, 15.0)	12.0 (3.7, 33.5)	15.3 (7.0, 33.0)
IFN-γ (pg/ml)	7.5 (2.6, 16.5)	3.5 (1.5, 13.3)	6.1 (0.9, 15.3)	14.3 (7.1, 15.3)	10.9 (4.5, 19.9)	10.7 (6.1, 25.0)
VEGF (pg/ml)	115.3 (59.5, 198.6)	82.6 (26.1, 181.0)	54.5 (14.0, 109.2)	229.2 (107.3, 385.0)	177.9 (122.3, 338.0)	197.3 (110.5, 319.2)
Flt3L (pg/ml)	3.2 (0.6, 17.6)	3.2 (0.6, 8.3)	0.6 (0.6, 3.2)	3.2 (0.6, 32.9)	3.2 (0.6, 18.0)	3.2 (0.6, 16.0)
Fractalkine (pg/ml)	63.0 (42.7, 105.8)	48.7 (24.6, 77.1)	55.8 (28.6, 87.2)	97.7 (42.1, 186.5)	78.3 (48.8, 129.3)	79.7 (42.8, 119.6)
PDGF (pg/ml)	3235.5 (1663.5, 5304.7)	2038.2 (1193.4, 3290.7)	1078.5 (293.1, 2431.6)	2398.9 (1127.7, 4752.2)	2211.9 (960.1, 3350.5)	656.5 (3.2, 2078.6)
Selectin (pg/ml)	63.6 (46.8, 81.6)	46.7 (33.5, 66.5)	71.5 (57.5, 93.1)	59.9 (42.3, 84.9)	44.2 (30.0, 58.7)	70.6 (54.1, 84.8)
sICAM1 (pg/ml)	64.5 (45.1, 80.4)	46.4 (36.1, 62.5)	77.6 (58.2, 99.4)	62.8 (47.4, 83.4)	46.8 (35.8, 65.2)	73.4 (57.0, 99.7)
sVCAM1 (pg/ml)	727.2 (578.8, 903.2)	517.4 (415.9, 706.4)	787.0 (687.0, 943.7)	795.1 (658.2, 894.8)	564.2 (456.8, 752.9)	824.1 (726.6, 1088.1)
Lactate (mg/dl)	-	-	2.8 (2.2, 3.4)	-	-	4.2 (3.6, 5.1)

"-" indicate no data. T_1 10 min after baseline, T_2 20 min after T_1, T_3 18 h after T_2, *Flt3L* Fms-like tyrosine kinase 3 ligand, *IFN-γ* interferon gamma, *IL* interleukin, *PDGF* platelet derived growth factor, *sICAM1* serum intercellular cell adhesion molecule 1, *sVCAM1* serum vascular cell adhesion molecule 1, *TNF* tumor necrosis factor, *VEGF* vascular endothelial growth factor

Table S3 Bivariate models by natural logarithm of biomarker adjusted by ES2 score

Biomarker	Coef.	*P*-value	95%CI
TNF-α			
T_1	0.43	0.00	0.38 – 0.48
T_2	0.36	0.00	0.31 – 0.42
T_3	0.31	0.00	0.20 – 0.42
Treat	-0.20	0.03	-0.38 – -0.03
ES2 score	0.01	0.01	0.00 – 0.02
IL-6			
T_1	0.30	0.00	0.15 – 0.45
T_2	0.56	0.00	0.35 – 0.77
T_3	3.22	0.00	2.84 – 3.60
Treat	-0.37	0.08	-0.79 – 0.04
ES2 score	0.02	0.04	0.01 – 0.04
IL-10			
T_1	0.75	0.00	0.47 – 1.03
T_2	1.79	0.00	1.40 – 2.17
T_3	2.32	0.00	1.94 – 2.70
Treat	0.09	0.73	-0.39 – 0.56
ES2 score	0.02	0.06	0.00 – 0.04
Flt3L			
T_1	-0.21	0.16	-0.51 – 0.08
T_2	-0.76	0.00	-1.08 – -0.44
T_3	-0.93	0.00	-1.25 – -0.60
Treat	-0.23	0.47	-0.86 – 0.40
ES2 score	0.01	0.60	-0.02 – 0.03
Fractalkine			
T_1	-0.13	0.06	-0.27 – 0.00
T_2	-0.45	0.00	-0.65 – -0.26
T_3	-0.34	0.00	-0.54 – -0.14
Treat	-0.35	0.02	-0.65 – -0.06
ES2 score	0.00	0.69	-0.02 – 0.01

PDGF			
T_1	0.07	0.46	-0.12 – 0.26
T_2	-0.46	0.00	-0.77 – -0.15
T_3	-1.97	0.00	-2.51 – -1.43
Treat	0.72	0.02	0.10 – 1.34
ES2 score	-0.02	0.22	-0.06 – 0.01
Selectin			
T_1	-0.08	0.00	-0.13 – -0.03
T_2	-0.40	0.00	-0.48 – -0.33
T_3	0.06	0.06	0.00 – 0.13
Treat	0.07	0.47	-0.12 – 0.27
ES2 score	0.00	0.46	-0.01 – 0.01
sICAM1			
T_1	-0.11	0.00	-0.14 – -0.08
T_2	-0.37	0.00	-0.41 – -0.33
T_3	0.09	0.02	0.01 – 0.17
Treat	-0.01	0.95	-0.17 – 0.15
ES2 score	0.00	0.90	-0.01 – 0.01
sVCAM1			
T_1	-0.07	0.00	-0.11 – -0.04
T_2	-0.36	0.00	-0.43 – -0.29
T_3	0.06	0.04	0.00 – 0.13
Treat	-0.04	0.45	-0.14 – 0.06
ES2 score	0.00	1.00	-0.01 – 0.01

Coef. regression coefficient, *95%CI* 95% confidence interval, *ES2* European System for Cardiac Operative Risk Evaluation, *IL* interleukin, *PDGF* platelet derived growth factor, *sICAM1* serum intercellular cell adhesion molecule 1, *sVCAM1* serum vascular cell adhesion molecule 1, *TNF* tumor necrosis factor, T_1 10 min after baseline, T_2 20 min after T_1, T_3 18 h after T_2, *VEGF* vascular endothelial growth factor

Table S4 Bivariate models on natural logarithm of TNF-α, PDGF and fractalkine adjusted by ES2 score and cytokine score at T_0

**Biomarker**	**Coef.**	***P*-value**	**95%CI**
TNF-α			
T_2	-0.07	0.00	-0.11 – -0.02
T_3	-0.12	0.02	-0.22 – -0.02
Treat	-0.03	0.49	-0.13 – 0.06
ES2 score	0.00	0.38	-0.01 – 0.00
TNF-α_T_0	0.80	0.00	0.71 – 0.89
PDGF			
T_2	-0.54	0.00	-0.80 – -0.27
T_3	-2.06	0.00	-2.60 – -1.51
Treat	0.20	0.24	-0.14 – 0.54
ES2 score	0.00	0.95	-0.02 – 0.01
PDGF_T_0	0.67	0.00	0.41 – 0.93
Fractalkine			
T_2	-0.33	0.00	-0.50 – -0.15
T_3	-0.20	0.04	-0.39 – -0.01
Treat	-0.37	0.01	-0.63 – -0.11
ES2 score	0.01	0.14	0.00 – 0.02
Fractalkine_T_0	0.73	0.00	0.57 – 0.89

PDGF platelet derived growth factor, *TNF* tumor necrosis factor, T_2 20 min after T_1, T_3 18 h after T_2

Table S5 Bivariate models on natural logarithm of IFN-γ, VEGF and lactate adjusted by ES2 score for treated (TCS10 group) and untreated (control group) patients

**Biomarker**	**Coef.**	***P*-value**	**95%CI**
IFN-γ			
Untreated			
T_1	0.06	0.63	-0.19 – 0.31
T_2	-0.61	**0.00**	-1.00 – -0.22
T_3	-0.15	0.5	-0.57 – 0.28
Treated			
T_1	-0.66	**0.00**	-1.01 – -0.30
T_2	-0.92	**0.00**	-1.26 – -0.59
T_3	-0.8	**0.00**	-1.22 – -0.38
ES2 score	0.01	0.69	-0.03 – 0.04
VEGF			
Untreated			
T_1	0.30	0.07	-0.02 – 0.61
T_2	0.14	0.52	-0.28 – 0.57
T_3	0.26	0.26	-0.19 – 0.71
Treated			
T_1	-0.68	**0.00**	-0.99 – -0.37
T_2	-0.97	**0.00**	-1.33 – -0.6
T_3	-1.53	**0.00**	-1.92 – -1.14
ES2 score	-0.01	0.94	-0.03 – 0.02
Lactate			
Untreated			
T_3	0.05	0.66	-0.17 – 0.26
Treated			
T_3	-0.22	**0.04**	-0.42 – -0.01
ES2 score	0.00	0.23	0.00 – 0.01

VEGF vascular endothelial growth factor, *IFN-γ* interferon gamma, *Coef.* regression coefficient, *95%CI* 95% confidence interval, *ES2* European System for Cardiac Operative Risk Evaluation, T_1 10 min after baseline, T_2 20 min after T_1, T_3 18 h after T_2

Table S6 Frequency of unavailable data (missing or degraded) according to biomarkers and time points and Mann-Whitney *U* test for testing MAR assumption

**Biomarker**	**Data (missing or degraded, *n*)**	**Mann-Whitney *U* test**		
		**T_1 vs. T_0**	**T_2 vs. T_1**	**T_3 vs. T_2**
IL-6				
T_0	3			
T_1	5			
T_2	3			
T_3	1			
IL-10				
T_0	9			
T_1	3			
T_2	2			
T_3	1			
IFN-γ				
T_0	0	0.001		
T_1	8		0.045^{**}	
T_2	5			
T_3	8			
VEGF				
T_0	1			
T_1	5			
T_2	4			
T_3	10			
Flt3L				
T_0	7			
T_1	10			
T_2	9			
T_3	11			
Fractalkine				
T_0	1	0.01^{**}		
T_1	4			

T_2	5		
T_3	3		
PDGF			
T_0	1		
T_1	2		0.01**
T_2	5		
T_3	13		
Selectin			
T_0	1	NA*	
T_1	1		
T_2	2		
T_3	5		
sICAM1			
T_0	2	NA*	
T_1	2		
T_2	3		
T_3	7		
sVCAM1			
T_0	2	NA*	
T_1	2		
T_2	3		
T_3	5		

As for TNF-α and lactate, no missing analysis was performed because only 1 missing value of these biomarkers were observed at T_3. *Bonferroni's correction is not applicable. **Not significant after Bonferroni correction. *Flt3L* Fms-like tyrosine kinase 3 ligand, *IFN-γ* interferon gamma, *IL* interleukin, *sICAM1* serum intercellular cell adhesion molecule 1, *sVCAM1* serum vascular cell adhesion molecule 1, *TNF* tumor necrosis factor, *PDGF* platelet derived growth factor, *VEGF* vascular endothelial growth factor, T_0 baseline, T_1 10 min after baseline, T_2 20 min after T_1, T_3 18 h after T_2, *MAR* missing at random, *NA* not available

Table S7 Logistic regression analyses of presence/absence of bleeding according to the allocation arm and stratified by relevant patients' strata

**Potential effect modifier**	***OR*(95%CI) (TCS10 vs. control)**	***P*-value (TCS10 vs. control)**	***P*-value for effect modification**
Age (years)			0.67
< median (75.5)	0.49 (0.16 – 1.56)	0.23	
> median (75.5)	0.70 (0.23 – 2.17)	0.54	
Gender			0.76
Female	0.67 (0.20 – 2.12)	0.49	
Male	0.52 (0.16 – 1.64)	0.26	
BMI (kg/m^2)			0.13
< median (26.1)	1.06 (0.35 – 3.19)	0.92	
> median (26.1)	0.30 (0.09 – 1.01)	**0.05**	
ES2 score			0.31
< median (20.5)	0.41 (0.13 – 1.27)	0.12	
> median (20.5)	0.82 (0.26 – 2.61)	0.74	
TNF-α (pg/ml)			0.46
< median (6.6)	0.48 (0.15 – 1.55)	0.22	
> median (6.6)	0.88 (0.28 – 2.81)	0.83	
IL-6 (pg/ml)			0.37
< median (0.9)	0.60 (0.24 – 1.48)	0.27	
> median (0.9)	0.63 (0.26 – 1.51)	0.30	
IL-10 (pg/ml)			0.65
< median (0.6)	0.49 (0.16 – 1.45)	0.20	
> median (0.6)	0.73 (0.19 – 2.81)	0.64	
IFN-γ (pg/ml)			0.20
< median (12.2)	0.98 (0.32 – 3.02)	0.97	
> median (12.2)	0.34 (0.10 – 1.08)	0.07	
VEGF (pg/ml)			0.78
< median (183.3)	0.55 (0.17 – 1.71)	0.30	
> median (183.3)	0.69 (0.21 – 2.25)	0.54	
Flt3L (pg/ml)			0.18

< median (9.1)	1.06 (0.34 – 3.36)	0.92	
> median (9.1)	0.34 (0.10 – 1.13)	0.08	
Fractalkine (pg/ml)			0.81
< median (91.1)	0.54 (0.17 – 1.72)	0.30	
> median (91.1)	0.66 (0.21 – 2.04)	0.47	
PDGF (pg/ml)			0.23
< median (2940)	1.00 (0.33 – 3.06)	1.00	
> median (2940)	0.36 (0.11 – 1.21)	0.10	
Selectin (pg/ml)			0.43
< median (63.3)	0.83 (0.27 – 2.53)	0.74	
> median (63.3)	0.43 (0.13 – 1.40)	0.16	
sICAM1 (pg/ml)			0.74
< median (69.5)	0.55 (0.18 – 1.73)	0.31	
> median (69.5)	0.73 (0.23 – 2.32)	0.59	
sVCAM1 (pg/ml)			0.44
< median (783.5)	0.87 (0.28 – 2.71)	0.80	
> median (783.5)	0.46 (0.14 – 1.45)	0.18	
Lactate (mg/dl)			0.60
< median (3.9)	0.47 (0.15 – 1.42)	0.18	
> median (3.9)	0.73 (0.22 – 2.39)	0.35	

BMI body mass index, *Flt3L* Fms-like tyrosine kinase 3 ligand, *IFN-γ* interferon gamma, *IL* interleukin, *sICAM1* serum intercellular cell adhesion molecule 1, *sVCAM1* serum vascular cell adhesion molecule 1, *TNF* tumor necrosis factor, *PDGF* platelet derived growth factor, *VEGF* vascular endothelial growth factor

Table S8 Bivariate logistic regression analyses of baseline biomarkers and treatment on bleeding

**Biomarkers and treatment**	***OR***	**95%CI**	***P*-value**
Log (TNF-α)	1.81	0.77 – 4.39	0.18
Treatment	0.64	0.28 – 1.46	0.29
Log (TNF-β)	0.94	0.49 – 1.81	0.84
Treatment	0.59	0.25 – 1.33	0.21
Log (IL-6)	1.88	1.37 – 2.77	0.00
Treatment	0.84	0.34 – 2.08	0.70
Log (IL-10)	1.50	1.15 – 2.08	0.01
Treatment	0.61	0.25 – 1.47	0.27
Log (IFN-γ)	0.82	0.60 – 1.11	0.21
Treatment	0.56	0.25 – 1.25	0.16
Log (VEGF)	0.86	0.61 – 1.17	0.35
Treatment	0.58	0.26 – 1.31	0.19
Log (fractalkine)	0.81	0.47 – 1.35	0.42
Treatment	0.58	0.25 – 1.30	0.19
Log (PDGF)	0.90	0.65 – 1.17	0.46
Treatment	0.65	0.28 – 1.47	0.30
Log (selectin)	1.43	0.66 – 3.19	0.36
Treatment	0.58	0.25 – 1.30	0.19
Log (sICAM1)	0.94	0.36 – 2.42	0.90
Treatment	0.63	0.28 – 1.41	0.27
Log (sVCAM1)	0.57	0.13 – 2.41	0.45
Treatment	0.63	0.28 – 1.41	0.27
Log (lactate)	3.06	1.44 – 7.17	0.01
Treatment	0.53	0.22 – 1.22	0.14

Flt3L Fms-like tyrosine kinase 3 ligand, *IFN-γ* interferon gamma, *IL* interleukin, *sICAM1* serum intercellular cell adhesion molecule 1, *sVCAM1* serum vascular cell adhesion molecule 1, *TNF* tumor necrosis factor, *PDGF* platelet derived growth factor, *VEGF* vascular endothelial growth factor

Table S9 Effect modification by baseline Flt3L (log transformed) on the relationship between treatment and bleeding

**Flt3L (quartiles of log transformed values)**	***OR***	**95%CI**	***P*-value**
1st log (Flt3L): -0.51	2.28	0.53 – 9.81	0.27
2nd log (Flt3L): 1.16	0.88	0.35 – 2.17	0.77
3rd log (Flt3L): 2.95	0.31	0.11 – 0.90	**0.03**

Flt3L Fms-like tyrosine kinase 3 ligand

Table S10 Univariate logistic regressions by cytokine at baseline on blood units transfused

Item	*OR*	95%CI	*P*-value
Log (TNF-α)	1.31	0.58 – 3.02	0.52
Log (TNF-β)	0.7o	0.29 – 1.31	0.32
Log (IL-6)	1.22	0.96 – 1.58	0.11
Log (IL-10)	1.11	0.88 – 1.40	0.38
Log (IFN-γ)	0.90	0.67 – 1.22	0.50
Log (VEGF)	0.79	0.56 – 1.07	0.15
Log (Flt3L)	0.87	0.68 – 1.11	0.26
Log (fractalkine)	0.95	0.57 – 1.59	0.86
Log (PDGF)	0.94	0.72 – 1.22	0.66
Log (selectin)	1.95	0.9 – 4.52	0.10
Log (sICAM1)	1.40	0.55 – 3.65	0.49
Log (sVCAM1)	1.88	0.45 – 8.18	0.39

Flt3L Fms-like tyrosine kinase 3 ligand, *IFN-γ* interferon gamma, *IL* interleukin, *sICAM1* serum intercellular cell adhesion molecule 1, *sVCAM1* serum vascular cell adhesion molecule 1, *TNF* tumor necrosis factor, *PDGF* platelet derived growth factor, *VEGF* vascular endothelial growth factor

Table S11 Bivariate logistic regression analyses of baseline biomarkers and treatment on transfusion.

Item	*OR*	95%CI	*P*-value
Log (TNF-α)	1.07	0.45 – 2.55	0.88
Treatment	0.33	0.14 – 0.74	0.01
Log (TNF-β)	0.60	0.23 – 1.17	0.19
Treatment	0.29	0.12 – 0.67	0.01
Log (IL-6)	1.17	0.91 – 1.52	0.22
Treatment	0.39	0.16 – 0.90	0.03
Log (IL-10)	1.08	0.85 – 1.37	0.54
Treatment	0.31	0.13 – 0.74	0.01
Log (IFN-γ)	0.87	0.63 – 1.18	0.37
Treatment	0.31	0.13 – 0.71	0.01
Log (VEGF)	0.81	0.56 – 1.12	0.22
Treatment	0.32	0.13 – 0.72	0.01
Log (Flt3L)	0.87	0.68 – 1.12	0.29
Treatment	0.34	0.14 – 0.80	0.01
Log (fractalkine)	0.87	0.51 – 1.47	0.59
Treatment	0.32	0.14 – 0.74	0.01
Log (PDGF)	1.02	0.76 – 1.33	0.91
Treatment	0.34	0.14 – 0.80	0.01
Log (selectin)	2.33	1.04 – 5.62	0.05
Treatment	0.29	0.12 – 0.67	0.00
Log (sICAM1)	1.53	0.58 – 4.19	0.40
Treatment	0.33	0.14 – 0.76	0.01
Log (sVCAM1)	2.03	0.46 – 9.34	0.35
Treatment	0.34	0.14 – 0.77	0.01
Log (lactate)	1.12	0.55 – 2.30	0.75
Treatment	0.32	0.14 – 0.72	0.01

Flt3L Fms-like tyrosine kinase 3 ligand, *IFN-γ* interferon gamma, *IL* interleukin, *sICAM1* serum intercellular cell adhesion molecule 1, *sVCAM* serum vascular cell adhesion molecule 1, *TNF* tumor necrosis factor, *PDGF* platelet derived growth factor, *VEGF* vascular endothelial growth factor
