# Supplementary material for: Effect of early administration of tetracosactide on mortality and host response in critically ill patients requiring rescue surgery: a sensitivity analysis of the STOPSHOCK phase 3 randomized controlled trial
Source: Mil Med Res. 2024 Aug 19;11:56. doi: 10.1186/s40779-024-00555-2 (PMC11331742; doi:10.1186/s40779-024-00555-2)
Supplement: Supplementary file 2 — Additional file 2. [file 40779_2024_555_MOESM2_ESM.pdf]

Pharmacokinetics of 10 mg TCS10 i.v. injection

TCS10 (or tetracosactide) is an analogous synthetic peptide that is identical to the 24-amino acid segment (sequence: SYSMEHFRWGKPVGKKRRPVKVYP) at the N-terminal of adrenocorticotropic hormone (ACTH) 1 – 24 (DrugBank online accession DB01284) [1]. The dose-strength and force analysis show that TCS10 has a molecular weight of 2933.5 Da and conversion for 10 mg = 1×10^{10} pg = 1×10^{7} ng = 0.001419 mmol = 1.419 pgmol/L = 1.419^{-12} mol/L.

The determination of safety in administering a single dose is observed by a half-life of 10 min and half-life clearance of 1.0 – 1.5 h in normal subjects. The distribution is 0.3 – 0.4 L/kg and there are no data in patients with hemodynamic complications.

The minimal anticipated biological effect level (MABEL)' approach is 7.0×10^{-9}/kg and active treatment dose of 5.4×10^{-9}/kg. Acute toxicity after intravenous administration in mice is LD_{50} = 190.29 mg/kg and in the dog is LD_{50} = 10 mg/kg and 30 mg/kg (i.e. negligible). In mice, 8-d of treatment showed an LD_{50} of 255 mg/kg and 8.7 mg/kg. The chronic administration toxicity study results in 0.3 mg/kg, 0.1 mg/kg, and 0.03 mg/kg after treatment 6 – 13 weeks. However, all regimens were tolerated. There were no cases of fatality. The no observed adverse effect level (NOAEL) by bovine serum albumin (BSA; mg/kg) in conversion factor, and the MABEL of 7 nmol/kg by active treatment dose [maximum recommended starting dose (MRSD)], is 5.4×10^{-9}/kg [2-4].

As mentioned, the target distribution workspace has a total body weight of approximately 1.2 kg and an area of approximately 5000 mq, equivalent to 14.228 mg/kg. The melanocortin receptor is calculated by signal *pathfinding* in blood volume CD18 density in expression target from 1.03×10^{-8} to 5.27×10^{-9} mol/L.

Human equivalent dose conversion allometric rat and dog by study dose and human equivalence are by respectively organism and sample dose of: study mice (approximately 20 g weight)/10.26 mg/kg (0.21 mg), human equivalent (approximately 60 kg weight)/10.26 mg/kg (615.6 mg) [1]. The potency dose of TCS10 is on maximal tolerated dose of Hi3 + 3 - up to ED_{50} receptor occupancy model and efficacy on pharmacodynamic biomarker assessment:

MABEL = 7.0×10^{-9}/kg active

MRSD = 5.4×10^{-9}/kg

NOAEL > 50.7×10^{-14} mol/(L·kg)

Bayesian sensitivity analysis

The association between severity markers, hazard function and the events death or alive were carried out using a two-stage approach for Bayesian joint model [5] of time to event failure rate in survival

function (Kaplan-Meier imputation, KMI) [6] and time to event risk rate of accelerated failure in a censored finite time.

The longitudinal process includes a mixed effects model for ES2 Z-score and a mixed effects beta regression model. The model indicates that ES2 Z-score for lifesaving emergency surgery and critical status is strongly related to survival function.

The sensitivity indexes were extrapolated by the Weibull shape parameter of the regression parametric regression model for survival data of risk set imputation and log-odds calibration for the hazard function, with historical data quoted by Z-area under receiver operating characteristic curve (Z-AUC) (ES2 score) [7,8].

Conceptually, we applied a Bhattacharyya's overlap measure of Fisher's exact test in a KMI [9-12], of Bayesian likelihood meta-analytic predictive prior informative of AUC curve and Euler-Cauchy equation for two-sample U-statistics in Bayesian paradigm as ES2 calibrated historical data.

Time to event failure rate by KMI

Time-to-event examines the duration until a predefined event occurs. The assumption in right censoring is the eventual event of life failure in evaluation methods like Kaplan-Meier, Cox regression and the log-rank test. The sensitivity approach is within the reference-based imputation based on the assumption that from the time point of their drop out onwards, patients who drop out of the active treatment group have the same risk of having an event as the patients in the reference group. For every pattern, the adjustments of any treatment to a predefined two-tailed δ that resulted in P-values < 0.05 was considered statistically significant. The continuous variables were expressed by mean and standard deviation (SD) and as M (Q_1, Q_3).

Patients' characteristics as pre-specified prior weight distributions of the linkage parameters between the two groups were compared by the Mann-Whitney U test. Under this assumption, KMI for the active treatment group is performed using the Kaplan-Meier curve of the reference group and control group as α-coefficient internal calibration. This represents a Cronbach's α (tau-equivalent) for internal consistency in R [13]. The cumulative credit rating for equality was calculated on a Lorenz curve distribution and Gini coefficient of meta-analytic predictive prior estimated pooled O/E ratio from a large sample size benchmark and coherence of the above-fraction in α-Lèvy probability distribution of R-squared independent variable in closed Kuder-Richardson Reliability Coefficients. The censoring at random method of multiple imputation via KMI represents a reference-based imputation in two-dimensional grid of models.

The combined stratum in KMI and factor analysis overlap the macro random censorship. The validity of bootstrap methods was investigated using real data for control type of error in a so-called power prior

approach that does not "borrow" the full historical information but uses a parameter $0 \leq \delta \leq 1$ to determine the amount of borrowed data (based on the methodology of the power prior, the frequentist framework allows incorporation of historical data from both groups of two-armed trials with binary outcome, while simultaneously controlling the type I error rate). It is shown that for any specific trial scenario a value $\delta > 0$ can be determined such that the type I error rate falls below the pre-specified significance level. The R-package implements a tipping point analysis of value δ depending on characteristics from treatment and external evidence via robust meta-analytic predictive priors. Sensitivity vs. specificity of calibrated Z-AUC score represents the observed-expected time-to event mortality informative. Conditionally on these characteristics, are the increase in power as compared to a trial without borrowing but require sample size reducing it.

Objective

The aim of this approach is to test if the difference in the survival curves of the active treatment group and the reference-calibrated group that it is still significant after benefit analysis of previously applied statistics. Finally, the Inferential statistics applied as a function of bootstrap were performed on a two-dimensional grid as robustness in factor analysis and modeling practice in existing guidelines [14-20]. The aim is to correctly detect the inflation of the type I error under the null parameter by compensating the probability weight with a negligible bias [16-19]. Key themes include quantifying sensitivity to different constraints, supporting assumptions for response rates in a small sample as a vector of finite population parameters.

Rationale

The framework is a proportional regression of the mean residual life of critically ill patients for aggregate survival data in reference-based imputation. The hierarchical time point is the accelerated lifetime failure through the proportional hazard dichotomous in state-space of the source population. Implementing the causal model requires untestable assumptions, so we need sensitivity analyses to understand the impact of these assumptions on inferences and conclusions from the primary analysis. Nonlinear Bayesian differential equations converge to time rapidly failing in tandem to shift the dependent curve. In this way, it is possible to estimate the likelihood density function in a time-dependent risk model. To obtain the solution, the non-homogeneous linear evolutionary equations reach the distribution of the Duhamel's in a bathtub curve. The response to an applied zero input and zero status is the fractional Tobit linear regression for a censored continuous result [11]. The central part of the curve is the half-life of the experimental drug and the continuous static failure rate with random sequence. The last part is stress-out breakdowns by accelerating the occurrence. The

probability free of events switch over occurrence to middle and final curve levels. The test improves estimates of care performance in case of postponement of the onset of the late failure phase. Otherwise, the accelerated failures will not occur in test time and coherence to the adaptive hypothesis.

The multi-state model and accelerated life expectancy failure in trial design architecture, enables data analysis to make joint probability of right censored data. Dynamic meta-analytic borrowing attains the operator of the Bayesian rule of maximum a posterior estimation. Furthermore, frequentist empirical likelihood-based approaches for Z-AUC estimation were converted in exact inferential procedure by adapting the empirical likelihood into a Bayesian framework and draw inference from the posterior samples of the Z-AUC obtained via a Gibbs sampler. The full conditional distributions within the Gibbs sampler only involve empirical likelihoods with linear constraints, which greatly simplify the computation and enhance the applicability and flexibility of the Bayesian empirical likelihood in comparison of multiple tests, and the doubly robust estimation. Weibull's conditioned survival gives parameters β shape and life characteristics ή, to increase the probability of distribution of the probability density and the final cumulative probability values. The hierarchical design-formula give the statistical cross-efficacy power of tau-equivalent reliability within various rank’s profiles of continuous mixed variable in α-Cronbach = 1. The gated time-weighted in principal strata, is the high-order attribution and rank-density cumulative distribution. The robust frame encodes coefficient Bayesian criterion of probability in reliability of Wigner’s matrices and coupled Hidden Markow chain. Survival analysis provided the equation in observed parameters within the stable linear α-Lèvy distribution to Gaussian fluctuation for linear statistics. The efficacious of investigated treatment, based on the total evidence provides an implementation of a graphical approach for different one- sided interval positive of the non-inferiority margin levels of 97.5% by above M_{source} and M_{weak} model [21]. The reconciliation of the formulation of the hypotheses and the calculation of type I error of two the calibrated critical value was obtained through meta analytic predictive prior that have a commensurate power prior for inferential analysis onto frequentist. The *t*-test sample gives the expected mortality rate and the previous probability of occurrence in the finite observational time domain. Power curves affect size in order of effect in an anterior distribution. Weighting is a computed power of the probability attributes of historical joints and the impact of uncertainty and degree of consistency in significance level. Under certain conditions, this sensitivity analysis increases power and supports a small sample size as a key subset compared to a two-stage approach test that does not require a large sample size.

References

[1] DrugBank. Tetracosactide. https://go.drugbank.com/drugs/DB01284. Accessed 2 Jan 2024.

[2] OnlineBiology. Gaurab Karki. G-protein coupled receptor (GPCR): β -adrenergic signalling pathway. 7 July 2020. https://www.onlinebiologynotes.com/g-protein-coupled-receptor-gpcr-β-adrenergic-signalling-pathway/. Accessed 2 Mar 2024.

[3] Ingersoll MA, Platt AM, Potteaux S, Randolph GJ. Monocyte trafficking in acute and chronic inflammation. Trends Immunol. 2011;32(10):470-7.

[4] European Medicines Agency. Guideline on setting health-based exposure limits for use in risk identification in the manufacture of different medicinal products in shared facilities. 20 Nov 2014. https://www.ema.europa.eu/en/documents/scientific-guideline/guideline-setting-health-based-exposure-limits-use-risk-identification-manufacture-different-medicinal-products-shared-facilities_en.pdf. Accessed 2 Mar 2024.

[5] Leiva-Yamaguchi V, Alvares D. A two-stage approach for Bayesian joint models of longitudinal and survival data: correcting bias with informative prior. Entropy (Basel). 2020;23(1):50.

[6] D'Arrigo G, Leonardis D, Abd ElHafeez S, Fusaro M, Tripepi G, Roumeliotis S. Methods to analyse time-to-event data: the Kaplan-Meier survival curve. Oxid Med Cell Longev. 2021;2021:2290120.

[7] Zhang Z. Parametric regression model for survival data: Weibull regression model as an example. Ann Transl Med. 2016;4(24):484.

[8] Jiang R, Murthy DNP. A study of Weibull shape parameter: properties and significance. Reliab Eng Syst Saf. 2011;96(12):1619-26.

[9] Gravestock I, Held L, COMBACTE-Net consortium. Adaptive power priors with empirical Bayes for clinical trials. Pharm Stat. 2017;16(5):349-60.

[10] Pan H, Yuan Y, Xia J. A calibrated power prior approach to borrow information from historical data with application to biosimilar clinical trials. J R Stat Soc Ser C Appl Stat. 2017;66(5):979-96.

[11] Lipkovich I, Ratitch B, O'Kelly M. Sensitivity to censored-at-random assumption in the analysis of time-to-event endpoints. Pharm Stat. 2016;15(3):216-29.

[12] Zhao Y, Herring AH, Zhou H, Ali MW, Koch GG. A multiple imputation method for sensitivity analyses of time-to-event data with possibly informative censoring. J Biopharm Stat. 2014;24(2):229-53.

[13] Allison A, White IR, Bond S. Rpsftm: an R package for rank preserving structural failure time models. R J. 2017;9(2):342-53.

[14] European Medicines Agency. CPMP/EWP/1776/99 Rev.1. https://www.ema.europa.eu/en/documents/scientific-guideline/guideline-missing-data-confirmatory-clinical-trials_en.pdf. Accessed 12 Nov 2023.

[15] Jin M, Fang Y. Methods for informative censoring in time-to-event data analysis. Stat Biopharm Res. 2024;16(1):47-54.

[16] Gorst-Rasmussen A, Tarp-Johansen MJ. Fast tipping point sensitivity analyses in clinical trials with missing continuous outcomes under multiple imputation. J Biopharm Stat. 2022;32(6):942-53.

[17] Feißt M, Krisam J, Kieser M. Incorporating historical two-arm data in clinical trials with binary outcome: a practical approach. Pharm Stat. 2020;19(5):662-78.

[18] Kuder GF, Richardson MW. The theory of the estimation of test reliability. Psychometrika. 1937;2(3):151-60.

[19] Nelson WB. Applied Life Data Analysis. John Wiley & Sons: New York, NY, USA, 1982. https://www.wiley.com/en-br/Applied+Life+Data+Analysis-p-9780471644620.

[20] Smith P. Analysis of failure and survival data. 1st edition. Chapman and Hall/CRC. 2002. https://www.routledge.com/Analysis-of-Failure-and-Survival-Data/Smith/p/book/9781584880752?gad_source=1&gclid=Cj0KCQjwpZWzBhC0ARIsACvjWRMMjCjFeKVT57GfK3J-_dK4HDVIiOSlbf7G33hdpPluOiGDe4T4apkaAsQCEALw_wcB.

[21] Banbeta A, van Rosmalen J, Dejardin D, Lesaffre E. Modified power prior with multiple historical trials for binary endpoints. Stat Med. 2019;38(7):1147-69.
